# Technical adequacy of bisulfite sequencing and pyrosequencing for detection of mitochondrial DNA methylation: Sources and avoidance of false-positive detection

**DOI:** 10.1371/journal.pone.0192722

**Published:** 2018-02-08

**Authors:** Chie Owa, Matthew Poulin, Liying Yan, Toshi Shioda

**Affiliations:** 1 Center for Cancer Research, Massachusetts General Hospital, Charlestown, MA, United States of America; 2 EpigenDx, Inc., Hopkinton, MA, United States of America; 3 Harvard Medical School, Boston, MA, United States of America; University of Perugia, ITALY

## Abstract

The existence of cytosine methylation in mammalian mitochondrial DNA (mtDNA) is a controversial subject. Because detection of DNA methylation depends on resistance of 5’-modified cytosines to bisulfite-catalyzed conversion to uracil, examined parameters that affect technical adequacy of mtDNA methylation analysis. Negative control amplicons (NCAs) devoid of cytosine methylation were amplified to cover the entire human or mouse mtDNA by long-range PCR. When the pyrosequencing template amplicons were gel-purified after bisulfite conversion, bisulfite pyrosequencing of NCAs did not detect significant levels of bisulfite-resistant cytosines (brCs) at ND1 (7 CpG sites) or CYTB (8 CpG sites) genes (CI_95_ = 0%-0.94%); without gel-purification, significant false-positive brCs were detected from NCAs (CI_95_ = 4.2%-6.8%). Bisulfite pyrosequencing of highly purified, linearized mtDNA isolated from human iPS cells or mouse liver detected significant brCs (~30%) in human ND1 gene when the sequencing primer was not selective in bisulfite-converted and unconverted templates. However, repeated experiments using a sequencing primer selective in bisulfite-converted templates almost completely (< 0.8%) suppressed brC detection, supporting the false-positive nature of brCs detected using the non-selective primer. Bisulfite-seq deep sequencing of linearized, gel-purified human mtDNA detected 9.4%-14.8% brCs for 9 CpG sites in ND1 gene. However, because all these brCs were associated with adjacent non-CpG brCs showing the same degrees of bisulfite resistance, DNA methylation in this mtDNA-encoded gene was not confirmed. Without linearization, data generated by bisulfite pyrosequencing or deep sequencing of purified mtDNA templates did not pass the quality control criteria. Shotgun bisulfite sequencing of human mtDNA detected extremely low levels of CpG methylation (<0.65%) over non-CpG methylation (<0.55%). Taken together, our study demonstrates that adequacy of mtDNA methylation analysis using methods dependent on bisulfite conversion needs to be established for each experiment, taking effects of incomplete bisulfite conversion and template impurity or topology into consideration.

## Introduction

Whether mitochondrial DNA (mtDNA) in mammalian cells is significantly methylated or not has been a highly controversial subject for over four decades [1]. Early studies using methylation-sensitive restriction assays observed up to 5% of CpG methylation in human and mouse mtDNA [2,3]. In contrast, Maekawa *et al*. reported inability of detecting bisulfite-resistant cytosines in mtDNA of human cancer tissues using the single-stranded DNA conformation polymorphism (SSCP) analysis [4]. The existence of 5-methylcytosines (5meCs) in mtDNA was later revived based on data obtained from a study using methylated DNA immunoprecipitation (MeDIP) and immunological detection of DNA methyltransferases DNMT1 [5] or DNMT3A [6] in the mitochondrial fraction. Using bisulfite pyrosequencing, several studies reported relatively high levels of mtDNA methylation [7–13]. However, bisulfite deep sequencing of purified human mtDNA fragments performed by Hong *et al*. did not detect appreciable levels of CpG methylation (≤ 0.66%) [14]. Liu *et al*. recently demonstrated that the closed-circular-covalent topology of human mtDNA effectively inhibits bisulfite conversion, resulting in significant false-positive detection of CpG methylation by bisulfite pyrosequencing [15]. The authors suggested that CpG methylation reported by studies performed without linearization of mtDNA [7,8,10] might be overestimation.

Overestimation of cytosine methylation is a commonly encountered technical problem, even for nuclear genomic DNA. Warnecke *et al*. reported common sources of false-positive detection of 5-methylcytosines using bisulfite pyrosequencing, demonstrating the impact of length, concentration, and purity of genomic DNA specimens to credibility of data [16]. Delancy *et al*. pointed the importance of single-band purity of PCR amplicons devoid of unincorporated primers for adequate specificity of bisulfite pyrosequencing [17]. The existence of a large number of nuclear integration of mtDNA sequences (NUMTs) further emphasizes the important of evaluating purity of mtDNA for CpG methylation analysis [12].

In the present study, we provide evidence that localized incompleteness of bisulfite conversion is a major source of false-positive detection of methylated cytosines in mtDNA. Using PCR amplicons of human and mouse mtDNA completely devoid of 5meCs and highly purified mtDNA isolated from human iPS cells and mouse liver, we show how mtDNA purity and topology impact false-positive detection of mtDNA methylation. We also show significant local heterogeneity in bisulfite conversion efficiency and propose strategies to evaluate and avoid artifacts derived from such heterogeneous incompleteness of bisulfite conversion. We conclude that adequate evaluation of mtDNA methylation should consider effects of local incompleteness of bisulfite conversion into consideration.

## Results

### False-positive detection of DNA methylation from negative-control PCR amplicons of human and mouse mtDNA by bisulfite pyrosequencing

**Fig 1:**
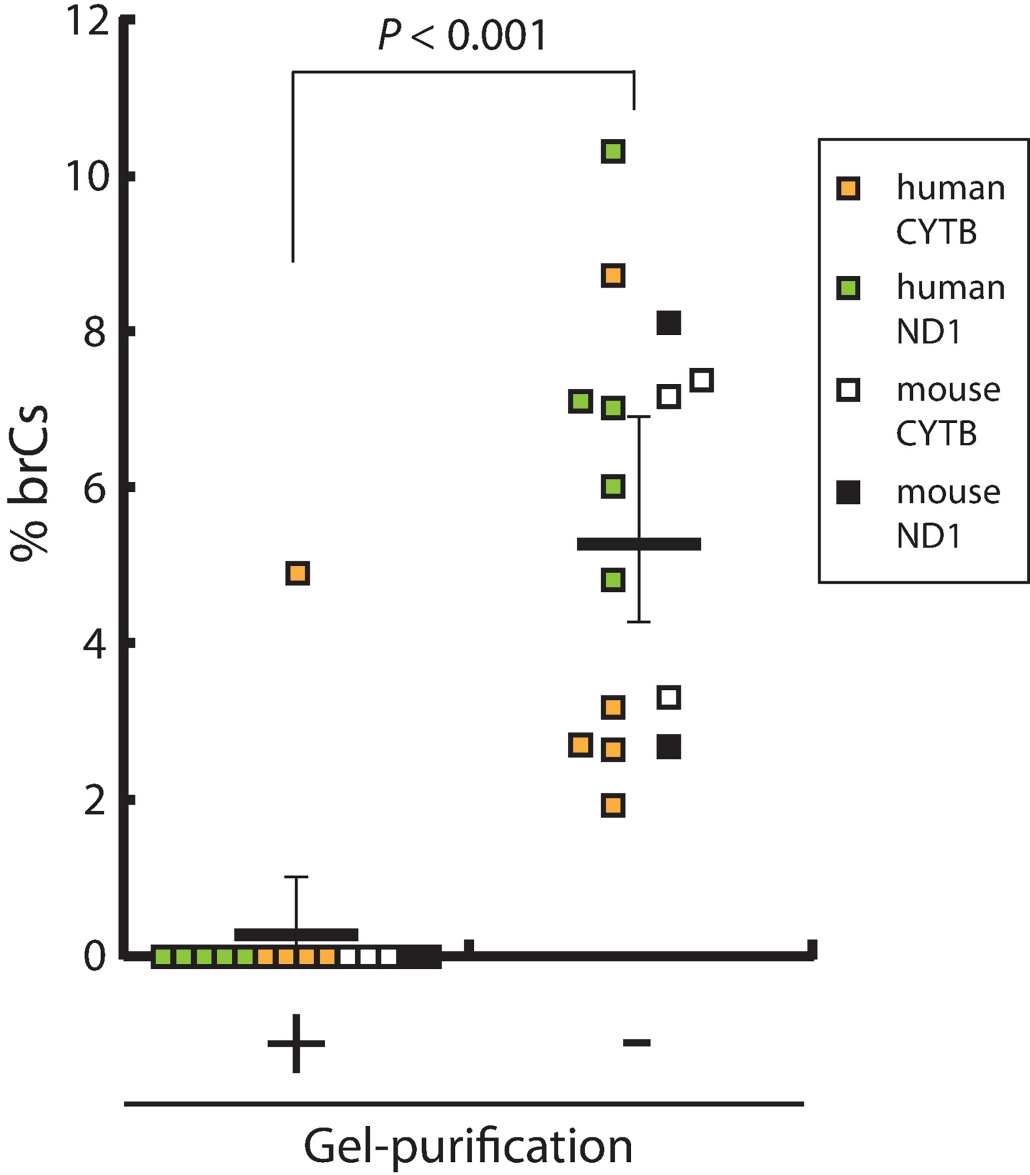
False-positive detection of bisulfite-resistant cytosines (brCs) in the negative control amplicons (NCAs) of mtDNA by bisulfite pyrosequencing. BrCs were detected in gel-purified or unpurified, bisulfite-converted PCR amplicons by pyrosequencing for 15 CpG sites: 5 in human ND1, 5 in human CYTB, 2 in mouse ND1, and 3 in mouse CYTB. The *p*-value was calculated using paired t-test (two-tails). The lines show mean ± 95% Confidence Intervals.

The existence of 5meC in mtDNA has been a highly controversial subject [1,15]. To obtain an initial insight into the technical adequacy of mtDNA methylation analysis, we attempted to confirm the absence of 5meC detection by bisulfite pyrosequencing from completely demethylated human and mouse mitochondrial genome. Total genomic DNA isolated from human iPS cells or mouse liver was subjected to long-range, high-fidelity PCR to obtain long amplicons that cover the entire mtDNA alongside each other (S1 Fig). After 30 cycles of PCR amplification, these amplicons were practically devoid of 5meCs (< 1/1.5^30^ = 5 x 10^−6^ even when amplification efficiency is as low as 1.5). Single-band amplicons (9.1kb and 11.2kb in human, 8kb and 8.6kb in mouse) were purified from agarose gel and designated as *negative control amplicons* (NCAs). To examine effects of DNA purity on efficiency of bisulfite conversion, pyrosequencing templates amplified from bisulfite-converted NCAs were prepared either before or after single-band gel purification. Following previous reports [7,10,15], we evaluated bisulfite conversion efficiency based on the C-to-U conversion of cytosines at non-CpG sites. We calculated ratios of pyrogram peak heights of converted cytosine (which was T) to total cytosine (C + T) and observed sufficiently high values before (CI_95_ = 98.23%-99.17%) and after (CI_95_ = 96.07%-99.79%) single-band gel purification. As expected, significant levels of bisulfite-resistant cytosines (brCs) were not detected at 14 CpG sites examined in the single-band purified NCAs (Fig 1), except for a single CpG assay showing substantial background noise. In contrast, in the absence of agarose gel purification of the NCAs after PCR, significant levels of brCs (1.9% - 10.3%) were detected at all 15 CpG sites (mean±SD = 5.5±2.7%, Fig 1; *p*<0.001, paired *t-*test against gel-purified assays). We next evaluated the linearity of bisulfite pyrosequencing detection of CpG methylation by preparing a series of mixtures of NCAs and *in vitro* methylated NCAs *without* post-PCR gel purification. The obtained correlation coefficients (Pearson’s R^2^) were greater than 0.98 for all 15 CpG sites examined; however, all regression lines crossed the y-axis significantly above the origin (S2 Fig). These observations suggest that significant amounts (up to 10%) of false-positive CpG methylation can be detected by bisulfite pyrosequencing in the absence of post-PCR agarose gel purification of amplicons produced from bisulfite-converted mtDNA templates. However, when CpG sites are strongly methylated beyond these background false-positive levels (>10%), bisulfite pyrosequencing assay shows excellent linearity up to 100% methylation.

### Effects of incomplete bisulfite conversion on detection of mtDNA methylation

Warnecke *et al*. reviewed sources of technical artifacts generated by bisulfite conversion-dependent DNA methylation and emphasized the importance of evaluating incomplete bisulfite conversion [16]. Such incompleteness may derive from various causes such as strand reannealing or impurity of the template DNA [16]. Within large regions of genomic DNA that show nearly complete efficiencies of bisulfite conversion, small regions with partially insufficient conversion of cytosines, both in the CpG and non-CpG contexts, are often recognized [16]. Although these observations were made primarily with nuclear genomic DNA, we presumed that localized incompleteness of bisulfite conversion may also be a significant source of false-positive detection of CpG methylation in mtDNA.

**Fig 2:**
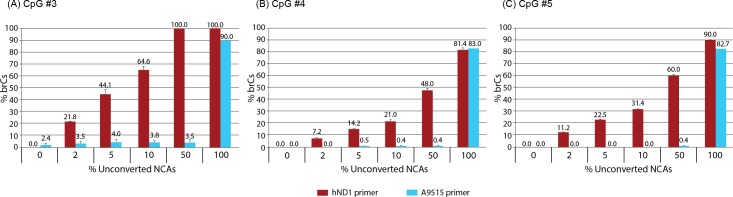
Effects of unconverted DNA on false-positive detection of CpG methylation. Mixtures of bisulfite-converted and unconverted NCAs were subjected to bisulfite pyrosequencing using sequencing primers A9515 or hND1. Each bar represents mean±SD of three independent analyses.

Bisulfite pyrosequencing assay of human ND1 gene in mtDNA (Fig 1) was performed using the sequencing primer originally designed by Liu *et al*., who demonstrated the importance of mtDNA linearization in DNA methylation analysis [15]. This primer (hND1 in Fig 2; originally designated as MT8 [15]) contained two bisulfite conversion-dependent annealing nucleotides–namely, the original cytosines (Cs) were replaced with thymidines (Ts)–and its 3’-end sequence was AAGA. We attempted to increase the selectivity of the sequencing primer within this assay to bisulfite-converted DNA templates by moving the target sequence towards the 3’ direction by 27 bp. The new sequencing primer (A9515 in Fig 2) contained four conversion-dependent annealing nucleotides, and its 3’-end sequence was AGTT, whose 3’-end TT dinucleotide was originally CC in the human genome reference sequence (GRCh38/hg38). Among the five CpG sites covered by the hND1 sequencing primer, three 3’-end CpG sites were interrogated by the A9515 primer. Annealing temperatures of hND1 and A9515 primers were estimated to be 49.8°C and 49.4°C, respectively. Details of primer design are shown in S3 Fig.

To evaluate selectivity of the hND1 and A9515 pyrosequencing primers to bisulfite-converted mtDNA templates, mixtures of bisulfite-converted and unconverted NCAs (not gel-purified after PCR amplification) were subjected to bisulfite pyrosequencing using these primers (Fig 2). To our surprise, the bisulfite pyrosequencing assay using the hND1 primer was extremely sensitive to the presence of very small amounts of unconverted NCA, and the impact of unconverted NCA remarkably differed between the CpG sites. When as small as 2% of the NCA template was unconverted, the bisulfite pyrosequencing assay estimated that over 20% of CpG #3 was methylated although the reason of this seemingly excessive false-positive detection is unknown. In contrast, the same assay using the A9515 primer effectively suppressed the false-positive impact of the presence of unconverted NCA occupying up to 50% of total NCA template (Fig 2; Assay pyrogram is shown in S4 Fig). When all NCA was unconverted template, bisulfite pyrosequencing using the A9515 primer technically failed (S4 Fig), resulting in very high false-positive values of DNA methylation (Fig 2). The strong selectivity of the A9515 primer to bisulfite-converted DNA templates was further supported by suppression of false-positive detection of 5meC at a non-CpG site (CpT) localized between the CpG sites 3 and 4, where the assay using the hND1 primer again estimated excessively strong cytosine methylation (S5 Fig). These results demonstrate the potentially very strong impact of incomplete bisulfite conversion to bisulfite pyrosequencing assay of mtDNA methylation as well as the existence of significant heterogeneity of such impact among individual CpG sites examined in a single assay. Our present study has confirmed importance of careful design of the pyrosequencing primer for suppression of the false-positive detection of mtDNA methylation caused by possible minor and/or location-dependent incompleteness of bisulfite conversion.

### Bisulfite pyrosequencing analysis of CpG methylation in mtDNA isolated from human and mouse cells

**Fig 3:**
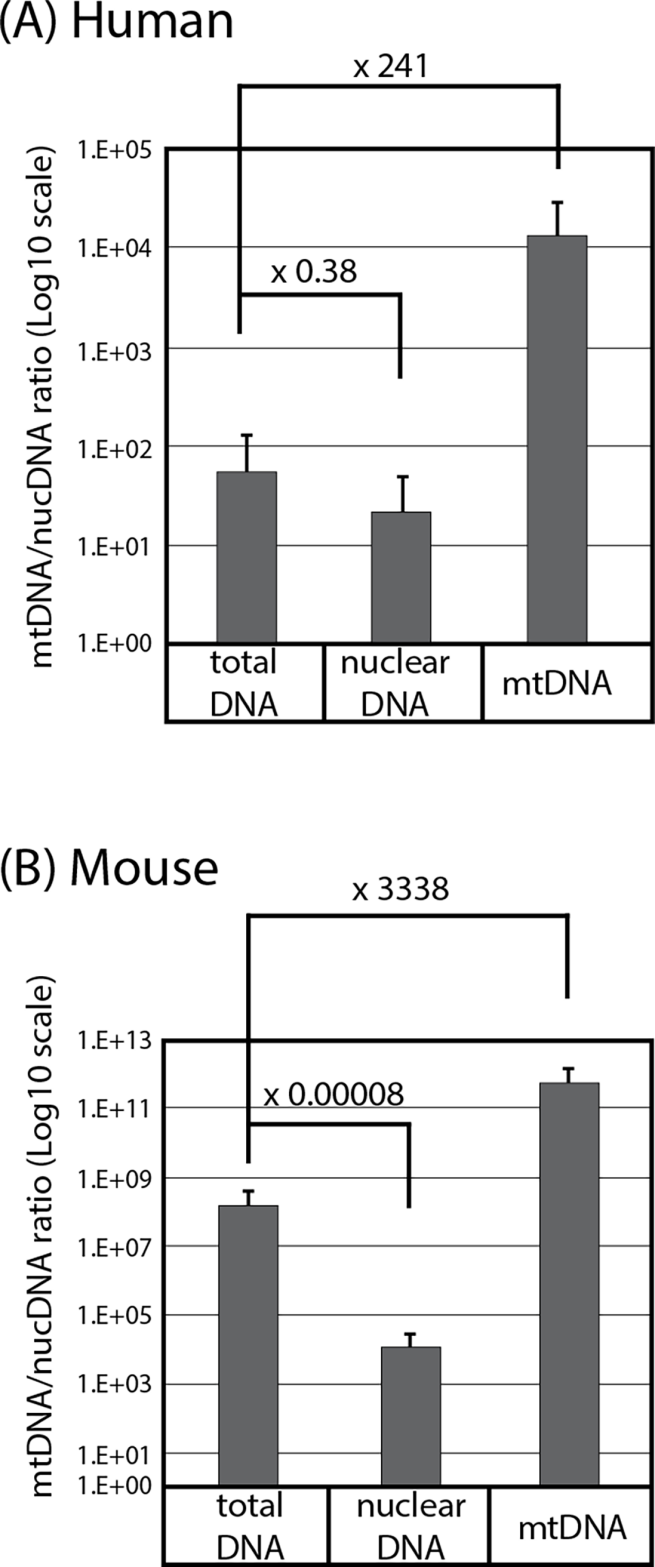
Enrichment of human and mouse mtDNA over nuclear DNA. Ratios of copy numbers of human (A) or mouse (B) mtDNA and nuclear DNA were determined by qPCR of mtDNA and nuclear DNA marker genes (Mean±SD, triplicated assays). Numbers show fold enrichment compared to total DNA.

Based on the strong impact of incomplete bisulfite conversion on a bisulfite pyrosequencing assay for CpG methylation demonstrated by using the synthetic mtDNA fragments, we next asked whether detection of CpG methylation in biological mtDNA specimens is also affected by selectivity of the pyrosequencing primers. To avoid possible false-positive detection of mtDNA methylation caused by contamination of nuclear genomic DNA [12], we purified human mtDNA isolated from iPS cell culture over 240-fold (Fig 3A; purity was evaluated by qPCR quantitation of genes encoded by nuclear and mtDNA genomes). Similarly, we isolated mouse mtDNA from liver tissues and purified over 3,000-fold (Fig 3B).

In agreement with the study performed by Liu *et al*. [15], our attempt to examine mtDNA methylation without linearizing the template did not generate pyrograms passing quality control criteria due to technical failure (S6 Fig). These results support the notion that the closed-circular-covalent topology of mtDNA suppresses bisulfite conversion and/or PCR amplification of the intact mtDNA template and that restriction digestion of mtDNA is critical for bisulfite pyrosequencing detection of mtDNA methylation [15].

**Fig 4:**
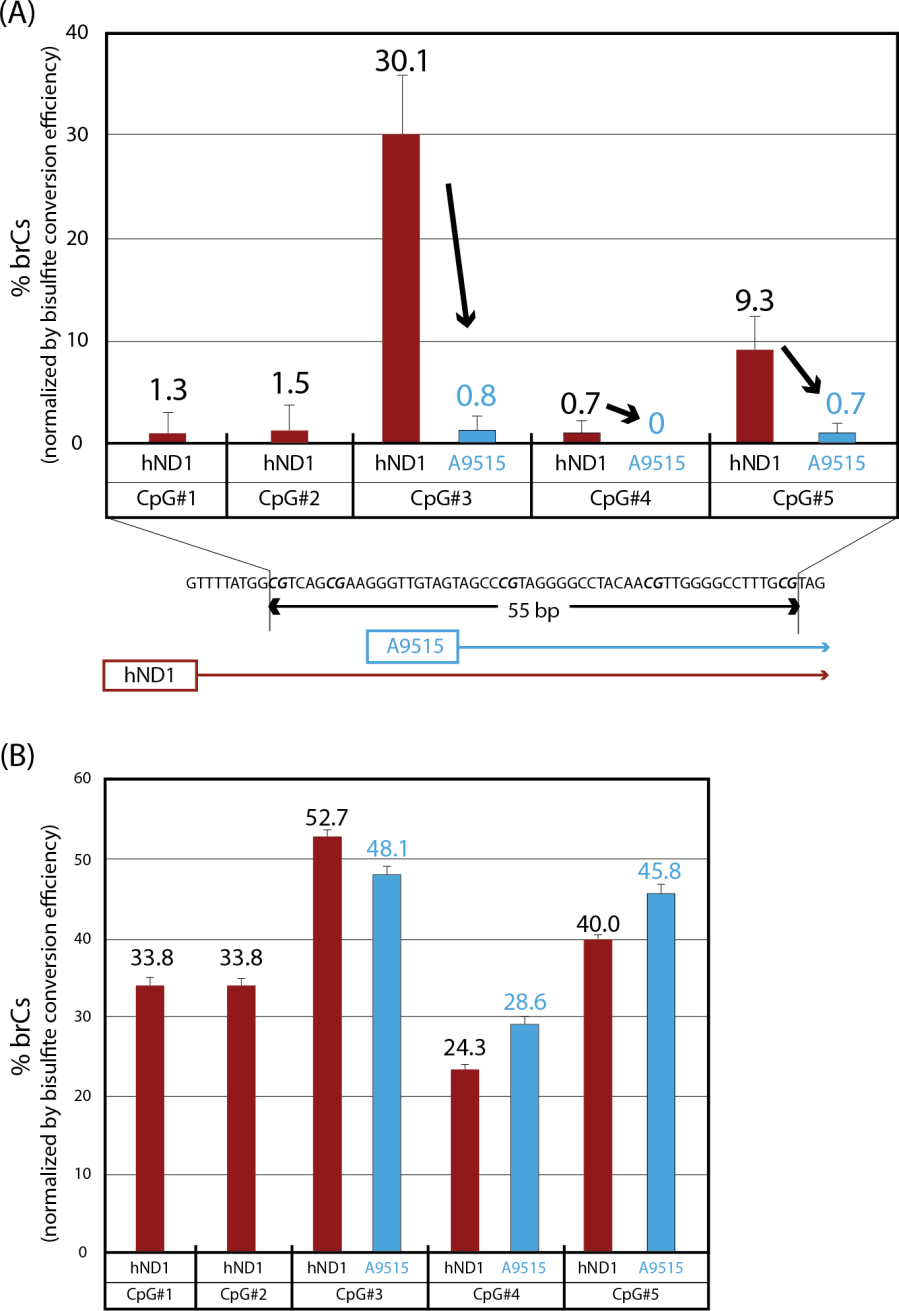
Detection of bisulfite-resistant cytosines in purified, linearized human mtDNA by bisulfite pyrosequencing using converted template-selective (A9515) and unselective (hND1) sequencing primers. Ratios of brCs were determined by bisulfite pyrosequencing (Mean±SD, triplicated assays). (A) The A9515 sequencing primer, which was highly selective to bisulfite-converted DNA, interrogated three CpG sites (CpG #3–5) whereas non-selective sequencing primer hND1 interrogated all these CpG sites plus two additional CpG sites (CpG #1 and 2). (B) Positive control assay was performed using *in vitro* partially methylated NCAs templates. High CpG methylation levels at three CpG sites (CpG #3–5) were detected using A9515 sequencing primer (CpG sites #1 and #2 were out of the assay coverage using this sequencing primer). hND1 sequencing primer detected high CpG methylation at all five CpG sites (CpG #1–5).

When human mtDNA was linearized by SphI digestion, purified using agarose gel, and subjected to bisulfite conversion, pyrosequencing using the hND1 sequencing primer detected significant CpG methylation in the ND1 gene (Fig 4A). Thus, among five CpG sites in a 55-bp window interrogated by this primer, CpG sites #3 and #5 showed as high as 30% and 9% brCs, respectively, while DNA methylation of the other three CpG sites was insignificant (<2%). However, pyrosequencing assay of the same, bisulfite-converted template mtDNA using the A9515 sequencing primer detected no significant CpG methylation at CpG sites #3, #4, or #5 (CpG sites #1 and #2 were out of the assay coverage using this sequencing primer).

Successful generation of clean pyrograms for the hND1 and A9515 sequencing primers is confirmed as shown in S7 Fig. As a positive control assay (*in vitro* partially methylated NCAs templates were used), A9515 sequencing primer detected high CpG methylation at the same levels of hND1 sequencing primer (Fig 4B). These results strongly suggest the false-positive nature of DNA methylation at CpG sites #3 and #5 in the ND1 gene of human mtDNA detected by the bisulfite pyrosequencing assay using the hND1 sequencing primer.

We also examined DNA methylation within the ND1 and CYTB genes in mouse mtDNA isolated from liver. After linearization by MluI digestion, mouse mtDNA was subjected to agarose gel purification and bisulfite conversion (efficiency >98%). The bisulfite pyrosequencing assay within the CYTB gene did not detect any 5meC in the three CpG sites interrogated by this assay (S8 Fig). The assay within the ND1 gene detected very low levels of 5meCs in the four interrogated CpG sites (2.7±2.0%, mean±SD) (S8 Fig).

### Targeted bisulfite deep sequencing analysis of mtDNA methylation

To obtain further insight into the technical adequacy of mtDNA methylation analysis based on methods dependent on bisulfite conversion, we examined cytosine methylation of human and mouse mtDNA by bisulfite sequencing targeting the ND1 and CYTB genes. Highly purified mtDNA isolate from human and mouse cells were linearized, purified using agarose gels, and bisulfite converted. The converted mtDNA templates were subjected to PCR amplification of the ND1 and CYTB genes, and the amplicons were sequenced using the Ion Torrent deep sequencer. Mixtures of unmethylated and enzymatically methylated NCAs were included in the procedure from the bisulfite conversion step as internal controls. Results of deep sequencing detection of mtDNA methylation are summarized in S1 Table.

**Fig 5:**
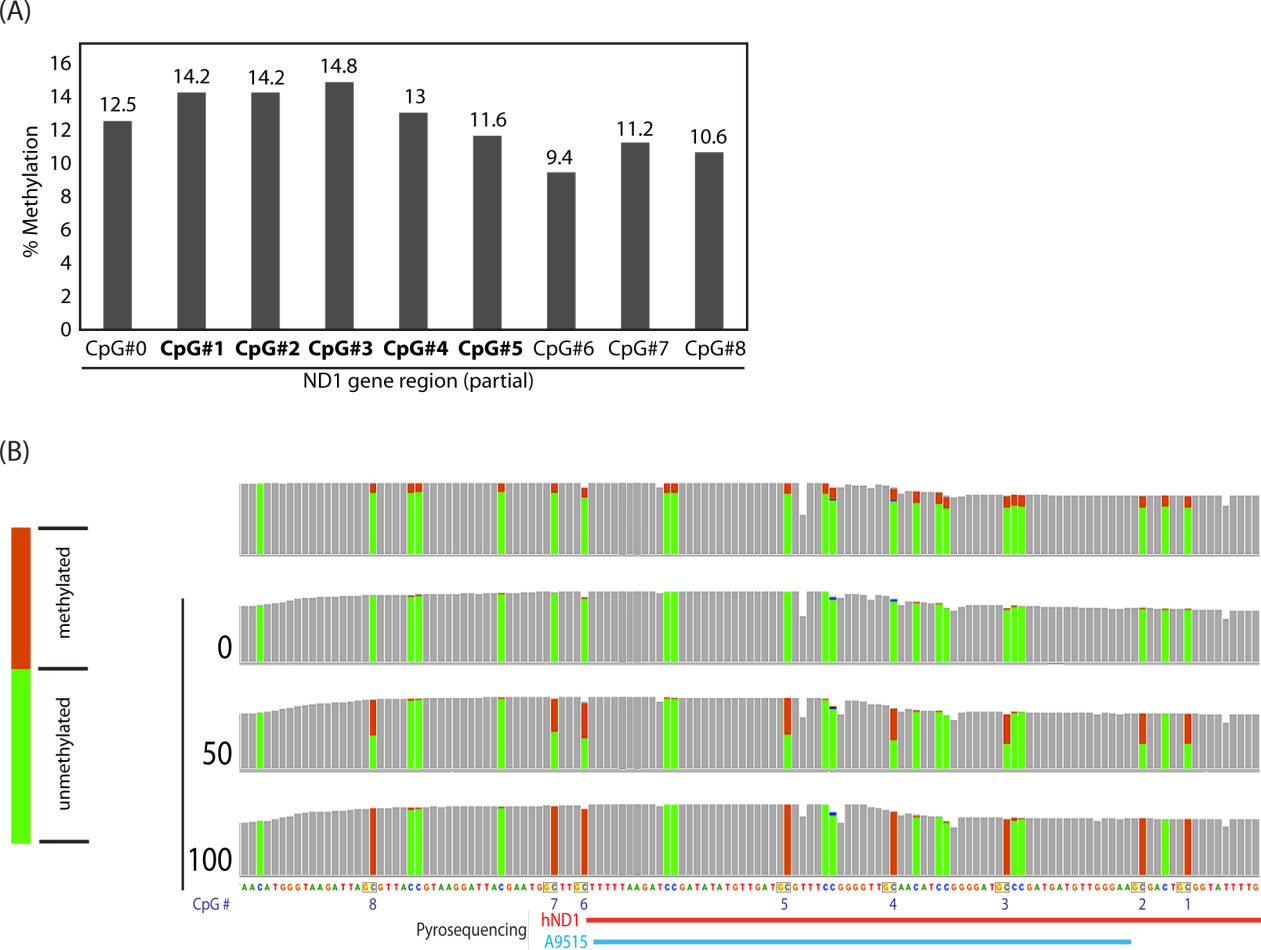
Targeted bisulfite deep sequencing of purified, linearized human mtDNA using hND1 primer. (A) Methylation of nine CpG sites in the mitochondrial ND1 gene determined based on the C/T SNP ratio. CpG sites #1 - #5 (shown in bold) correspond to the same names of CpG sites shown in Fig 4. (B) Deep sequencing coverage tracks showing C/T SNPs at CpG sites #0 - #8 indicated in panel A. CpG and non-CpG cytosines are indicated by red and blue arrows, respectively. Pileup coverages of C/T SNPs are indicated with brown C (unconverted) and green T (converted) bars, respectively.

Agreeing with the results of bisulfite pyrosequencing described earlier as well as a previously reported study of deep sequencing analysis of mtDNA methylation [14], targeted bisulfite-seq detection of human mtDNA methylation within the ND1 gene without template linearization resulted in technical failure, generating insufficient numbers of uniquely mapped reads (58–83 reads covering each CpG site). Modest numbers of reads (773–811 reads per CpG site) were generated for the CYTB gene from human mtDNA without linearization, suggesting that strength of the negative impact of the mtDNA topology to targeted bisulfite sequencing is not homogenous throughout the entire mitochondrial genome. Using a linearized mtDNA template, target bisulfite-seq analysis of the human ND1 gene detected 9.4–14.8% at nine consecutive CpG sites calculated based on the ratio of brCs (reflecting unconverted cytosines) and Ts (reflecting converted cytosines) (Fig 5A). All of these CpG sites were covered with 2,300–3,000 reads, supporting highly quantitative determination of CpG methylation. However, visualization of the deep sequencing data using the *Integrative Genomics Viewer* [18] revealed that not only the CpG cytosines but also all cytosines in the non-CpG contexts in the vicinity of these CpG sites showed exactly the same levels of brCs (Fig 5B). This characteristics is distinct from the confirmed non-CpG methylation of cytosines observed in biological specimens of nuclear genomic DNA, which typically show significantly stronger methylation at CpG dinucleotides compared to non-CpG contexts [19,20]. Therefore, we presume that the observed significant DNA methylation detected by targeted bisulfite sequencing in the human ND1 gene is likely false-positive detection caused by incomplete bisulfite conversion. In contrast, the same analytical approach did not detect significant levels of DNA methylation in the human CYTB gene (6 CpG sites) or the mouse ND1 or CYTB genes (7 and 3 CpG sites, respectively) although sufficient numbers of reads covered all of these CpG sites (806–2355 reads per CpG for ND1, and 2324–2431 reads for CYTB).

### Shotgun bisulfite deep sequencing analysis of mtDNA methylation

**Fig 6:**
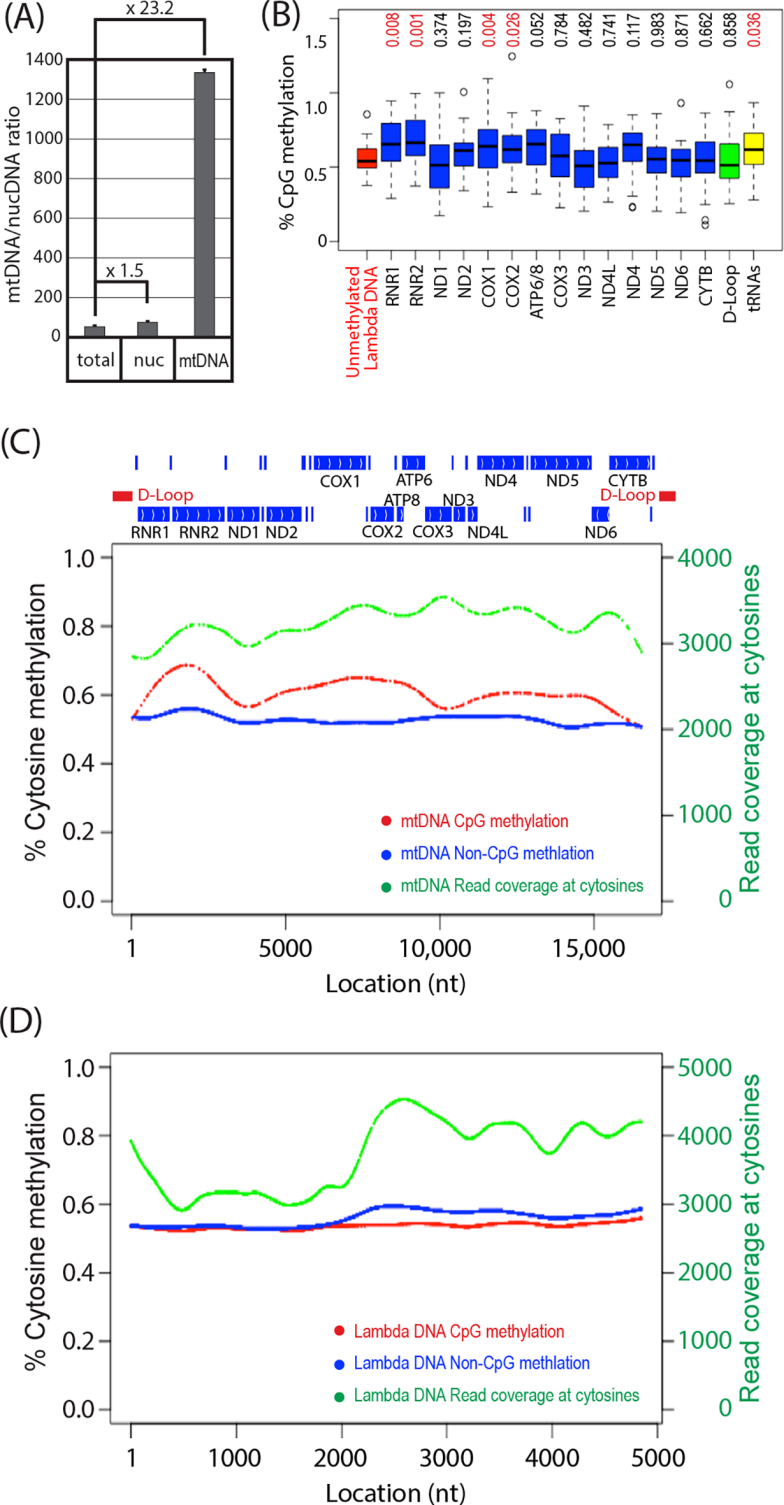
Shotgun bisulfite sequencing of human iPSC mtDNA. (A) Ratios of copy numbers of human mtDNA and nuclear DNA were determined by qPCR of mtDNA and nuclear DNA marker genes (Mean±SD, triplicated assays). Numbers show fold enrichment compared to total DNA. (B) CpG methylation of mtDNA-encoded genes (box plot; boxes and whiskers indicate quartiles and minimum/maximum values, horizontal lines in boxes indicate median). Numbers at the top indicate *p*-values of 2-tailed t-test against unmethylated lambda DNA. (C, D) Cytosine methylation of mtDNA (C) and unmethylated lambda DNA (D). Percentage of cytosine methylation in the CpG and non-CpG contexts is shown with red and blue dots, respectively. Deep sequencing read coverage at cytosines is shown with green dots. In panel (C), locations of mtDNA-encoded genes and D-loop are indicated at the top, where positions of tRNA genes are shown with vertical bars without gene names.

To determine cytosine methylation in the full-length mtDNA, we performed shotgun bisulfite sequencing of purified mtDNA isolated from human iPSCs. Because the shotgun sequencing procedure involves sonication of target DNA, we omitted enzymatic linearization of mtDNA. Gel purification of mtDNA was also omitted because mtDNA was not readily visualized without linearization. Thus, mtDNA was enriched from total cellular DNA by 23.2-fold (Fig 6A), sonicated to about 200 bp in length, and mixed with equimolar of sonicated, unmethylated lambda DNA. The mtDNA/lambda DNA mixture was subjected to shotgun sequencing with or without bisulfite conversion. In the absence of bisulfite conversion, most cytosines in CpG dinucleotides (>99.5% for mtDNA, >99.8% in lambda DNA) were correctly detected as cytosines in both mtDNA and lambda DNA (S10 Fig); in the presence of conversion, most cytosines were converted to thymidines (S9 Fig). No apparent regional bias in bisulfite conversion efficiency was observed (S9 Fig).

In all mtDNA-encoded genes and lambda DNA, CpG methylation did not exceed 1.0%, except for three CpG sites in ND2, COX2, and D-Loop (Fig 6B). None of these three CpG sites with greater than 1.0% methylation was associated with high cytosine methylation in non-CpG contexts. Compared to lambda DNA methylation, CpG methylation of RNR1, RNR2, COX1, COX2, and tRNA genes show statistically significant increase, although their absolute methylation levels were around 0.50%-0.75%. Low and consistent levels of cytosine methylation in non-CpG context (0.50%-0.55%) was detected throughout the length of mtDNA, whereas cytosine methylation at CpG sites showed region-dependent increase over non-CpG methylation to up to 0.65% (Fig 6C). In contrast, lambda DNA methylation at both CpG and non-CpG sites were nearly constant at 0.55%-0.60%. Detection of each cytosine methylation was based on at least 3000 uniquely mapped deep sequencing reads (Fig 6C and 6D). These results indicate that, in human iPSC mtDNA, extremely low levels of CpG methylation (up to 0.10% over 0.55% non-CpG methylation) can be detected without associating increase in non-CpG cytosine methylation. In unmethylated lambda DNA, approximately 0.5% cytosine methylation was detected in both CpG and non-CpG contexts, indicating that sensitivity limit of bisulfite shotgun sequencing detection of cytosine methylation with thousands of nucleotide-base coverage is approximately 0.5%.

## Discussion

The fundamental pitfall of various DNA methylation assays that are dependent on bisulfite conversion of cytosines to uracils is that detection of methylated cytosines (or, more broadly, cytosines whose 5-carbon is covalently modified with methyl, hydroxymethyl, formyl, or carboxyl groups) as brCs is indistinguishable from artifacts caused by incomplete C-to-U conversion [16]. Because bisulfite conversion occurs only in single-stranded DNA, incomplete denaturation of double-stranded DNA or re-annealing of dissociated DNA caused by repetitive sequences (such as Alu or LINE-1) and/or high DNA concentrations have been considered potential sources of false-positive artifacts of DNA methylation assays [16]. The closed-circular topology of mtDNA renders substantial resistance to bisulfite conversion without restriction digestion [15], and the presence of the short direct repeat sequences in human and mouse mtDNA (6–13 bp) [21] as well as multiple genes encoding transfer RNAs might facilitate re-annealing of dissociated mtDNA strands. Because the reported levels of mtDNA methylation are relatively low (typically not greater than 20%), potential false-positive artifacts derived from incomplete bisulfite conversion may significantly impact the biological interpretation of assay outcomes.

Mammalian mtDNA methylation has been a highly controversial subject for over four decades [1]. Table 1 summarizes recent studies on DNA methylation of mammalian mtDNA using methods dependent on bisulfite conversion of unmodified cytosines. Our present study has shown that the purity of bisulfite-converted pyrosequencing templates (Fig 1), presence of incompletely converted DNA in pyrosequencing reactions (Figs 2, 4 and 5), as well as topology of mtDNA before bisulfite conversion (S6 Fig and S1 Table) significantly affect the adequacy of DNA methylation analysis.

**Table 1:** Published studies on methylation of mammalian mtDNA determined using bisulfite pyrosequencing or deep sequencing.

Ref.	Method	mtDNA linearization	Evaluation of bisulfite conversion efficiency	mtDNA methylation
[7]	Bisulfite PyroSeq.	No	Non-CpG cytosine methylation (conversion rate not provided)	Yes (2–18%)
[8]	Bisulfite PyroSeq.	No	Not provided	Yes (1.6–6.5%)
[9]	Bisulfite PyroSeq.	No	Not provided	Yes (12–13%)
[10]	Bisulfite PyroSeq.	No	Non-CpG cytosine methylation (conversion rate not provided)	Yes (3.7–11.7%)
[22]	Bisulfite Deep Seq.	No	Not provided	Yes (CpG cytosines = 16–20%; All cytosines = 32–37%)
[14]	Bisulfite Deep Seq.	Yes (human; HindIII digestion)	Non-CpG cytosine methylation (not greater than 0.18%)	No (≤0.66%)
[15]	Bisulfite PyroSeq.	Yes (human; BamHI digestion)	Non-CpG cytosine methylation (conversion rate not provided)	Rare (<2%)

In agreement with the recent study of Liu *et al*. [15], enzymatic linearization of the mtDNA template was critical in our mtDNA methylation assays using both pyrosequencing and deep sequencing. This is apparently due to the resistance of the closed-circular topology of mtDNA to bisulfite conversion. Although the reason for the modest amounts of deep sequencing reads generated within the human CYTB gene without mtDNA linearization is not clarified (S1 Table), we speculate that it is the relatively short distance of the CYTB gene from the displacement loop (D-loop) structure (S1 Fig), where the double-stranded mtDNA is locally dissociated due to the presence of the third strand DNA, could facilitate dissociation of the mtDNA segment encoding this gene. In contrast, the mtDNA segment encoding the ND1 gene, which is distant from the D-loop (S1 Fig), could be more resistant to the alkaline dissociation process of bisulfite conversion reaction, resulting in poor generation of deep sequencing reads (S1 Table) or false-positive detection of DNA methylation (Fig 5). It is also interesting to speculate that replication, transcription, and damages of mtDNA could also affect bisulfite conversion efficiencies of non-linearized mtDNA specimens in a region-specific manner. Further studies will be necessary to determine how local displacement of the double-stranded mtDNA caused by the D-loop formation, mtDNA replication or transcription, or mtDNA damages caused by diseases or environmental factors, affects bisulfite conversion efficiencies.

Our present study suggests the existence of localized incompleteness of bisulfite conversion in mtDNA (Figs 2 and 4), which would result in false-positive detection of CpG methylation. Apparently, the conventional spike-in control of single-stranded or short double-stranded oligonucleotides for evaluation of bisulfite conversion efficiency cannot evaluate degrees of this type of false-positive artifacts. Although the spike-in controls have been successfully used for evaluation of DNA methylation in nuclear genomic DNA, this approach may not be optimal for the detection of reportedly weak DNA methylation in the mitochondrial genome, whose dissociation-annealing thermodynamic behavior may significantly differ from the nuclear genome. We therefore recommend evaluation of bisulfite conversion efficiencies based on brC detection in the non-CpG sites in the close vicinities of the target CpG sites of biological interest. It is also recommended to design pyrosequencing sequencing primers (i.e., primers to read nucleotide base sequence of amplified fragments) selective for bisulfite-converted DNA templates, which can effectively suppress false-positive detection of CpG methylation caused by the presence of unconverted DNA (Figs 2 and 4). It is our experience that sequencing primers with 3’-end TT dinucleotide targeting CC sequence often work well for this purpose. In addition to the sequencing primers for pyrosequencing reaction, PCR amplification primers involved in the bisulfite pyrosequencing procedure also need to be designed carefully. Fuso *et al*. reported that amplification primers designed using the MethPrimers may cause underestimation of CpG methylation because the software assumes that cytosines at non-CpG sites are mostly demethylated [23]. The authors proposed that, in the presence of significant amounts of physiological non-CpG cytosine methylation, methylation-insensitive primer design for amplification of bisulfite-converted DNA should be considered for suppress false-negatives [23]. Our amplification primer (assay A9515, forward primer; S3 Fig) was designed for preferential, but not exclusive, amplification of mtDNA with possible non-CpG methylation. Bisulfite pyrosequencing with a sequencing primer harboring a 3’-end TT dinucleotide did not detect methylated CpG in the A9515 amplicon of mtDNA isolated from human iPSCs (Fig 4A). When CpG sites are enzymatically methylated in the NCA using the spiroplasma M.SssI CpG methyltransferase *in vitro*, assay A9515 readily detected methylcytosines at CpG#3–5 of human mitochondrial ND1 gene (Fig 4B). In the absence of *in vitro* CpG methylation, the same assay did not detect CpG methylation in the NCA (Fig 2). These results support that the absence of CpG methylation in human iPSC mtDNA indicated by our assay A9515 was unlikely caused by underestimation of CpG methylation in DNA fragments containing non-CpG cytosine methylation.

Although the reason for the exaggerated false-positive detection of methylated CpG in the presence of relatively modest amounts of unconverted DNA in the pyrosequencing reaction is unknown (*e*.*g*., Fig 2A; the presence of 2% unconverted DNA resulted in 21.8% false positive detection of CpG methylation), we speculate that bisulfite-converted DNA could harbor significant damages in the DNA structure (such as strand breaks or depurination) and so are poor templates for PCR amplification before pyrosequencing, reducing the *effective* amounts of the bisulfite-converted template DNA that are suited to PCR amplification. Since complete bisulfite conversion of template DNA may be associated with the accumulation of partially damaged DNA, further studies may be required to establish optimized conditions of bisulfite conversion for mtDNA.

Evaluation of bisulfite conversion efficiency of mtDNA using brC levels at non-CpG sites is depending on an assumption that mtDNA methylation is specific to CpG sites. Because 5meCs detected at non-CpG sites in the nuclear genomes of embryonic stem cells, germline cells, or brain typically shows strong CpG methylation associated with significantly lower levels of non-CpG methylation [19,20], we presume that our deep sequencing detection of cytosine methylation with equal strength for all CpG and non-CpG sites in human ND1 gene was likely reflecting false-positive artifact rather than a biological phenomenon. Although a previous study observed non-CpG DNA methylation in human and mouse mtDNA [22], it would be necessary to examine the bisulfite conversion efficiency of their protocol due to the absence of mtDNA linearization in the description of their experimental procedure.

Our shotgun bisulfite sequencing analysis of purified human iPSC mtDNA detected region-dependent CpG methylation over non-CpG cytosine methylation (Fig 6C). However, the strength of CpG methylation was extremely weak (0.55%-0.65%) and comparable to non-CpG methylation (0.50%-0.55%). Our observations agree with a study reported by Hong *et al*., who detected up to 0.66% CpG methylation in mtDNA of human colon cancer cell lines using shotgun bisulfite sequencing [14]. Spike-in control lambda DNA, which was totally unmethylated due to the lack of DNA methyltransferase genes in the host bacteria, showed region-independent cytosine methylation at comparable levels (0.50%-0.60%) in both CpG and non-CpG contexts, indicating that detection of the CpG methylation in mtDNA was at the margin of the technical detection limit. It is unknown as to whether such extremely low levels of CpG methylation truly reflect the existence of methylcytosines in mtDNA, or they could be due to low-level contamination of CpG-methylated NUMTs. Because of limitation of the short-length deep sequencing, it is technically challenging to distinguish NUMT-derived CpG methylation signal and mtDNA CpG methylation.

In conclusion, our present study has shown the effects of mtDNA purity, topology, and local bisulfite conversion efficiency on the adequacy of DNA methylation assays. To suppress false-positive artifacts, we recommend the use of pyrosequencing primers highly selective to converted DNA templates. Taking the possible localized incompleteness of mtDNA bisulfite conversion, we recommend evaluation of conversion efficiencies based on brC levels of non-CpG cytosines in the close vicinities of the target CpG sites. Shotgun bisulfite sequencing detected extremely low levels (~0.5%) of region-dependent CpG methylation over non-CpG cytosine methylation in human iPSC mtDNA, whereas distinguishing true mtDNA methylation from effects of minuscule amounts of NUMT contamination is technically challenging. Future studies will be necessary to determine the exact levels of DNA methylation in the mitochondrial genome, which could show tissue specificity and/or affected by diseases or environmental factors.

## Materials and methods

### Preparation of the negative control amplicons (NCAs) representing demethylated mtDNA fragments

Total human DNA (consisting of nuclear DNA and mtDNA) was isolated from human iPS cells (clone A4 established in our lab) using Allprep Mini Kit (QIAGEN). Total mouse DNA was isolated from C57BL/6 mouse liver (Charles River) using DNeasy Blood and Tissue Kit (Qiagen). The NCAs were prepared from total DNA using Q5 High-Fidelity DNA Polymerase (New England Biolabs) by 30-cycle amplification (primers and PCR conditions are shown in S1 Fig) [24]. Single-band amplicons (9.2 and 11.2 kbp for human, 8.0 and 8.6 kbp for mouse) were excised from agarose gel after electrophoresis (0.7% SeaKem GTG agarose; Lonza) and purified using QIAquick gel extraction kit (QIAGEN) (S1 Fig).

### Purification of mtDNA and nuclear DNA

Human and mouse mtDNA and nuclear DNA was isolated from 50 million human iPS cells (clone A4) or 30 mg fresh liver tissue of C57BL/6 mice using Mitochondrial DNA Isolation Kit (abcam) following the manufacturer’s instruction. Human and mouse mtDNA was linearized by restriction digestion at unique sites using SphI-HF or MluI respectively, (New England Biolabs) and subjected to agarose gel purification to isolate the ~16.5 kbp DNA band. Purity of human mtDNA was evaluated by qPCR detection of mtDNA markers (MT-ND1 and MT-ND5) and nuclear DNA markers (SLCO2B1 and SERPINA1) using Human mtDNA Monitoring Primer Set (Cat# 7246; Takara Bio). Purity of mouse mtDNA was evaluated by qPCR quantitation of mtDNA marker MT-ND1 (Mm04225274-s1) and nuclear DNA marker *tert1* (Cat# 4403316) using Taqman Assays (Life Technologies).

### Bisulfite pyrosequencing

The NCAs and purified mtDNA specimens (with or without linearization) were subjected to bisulfite conversion using EZ DNA Methylation Kit (Zymo Research) following the manufacturer’s instructions. Pyrosequencing templates targeting MT-ND1 (mtDNA-encoded NADH ubiquinone oxidoreductase core subunit 1) and MT-CYTB (mtDNA-encoded cytochrome b) were amplified from bisulfite-converted NCAs using primers shown in S3 Fig (reverse primers were biotinylated) and HotStarTaq DNA Polymerase (QIAGEN). Thermal cycler was programmed for 95°C, 10 min; 40 cycles of 95°C for 30 sec, annealed for 40 sec, and 72°C for 60 sec; 72°C for 7 min (annealing temperatures: human ND1, 50°C, human CYTB, 51°C, mouse ND1, 52°C, mouse CYTB, 51°C). Where indicated, PCR amplicons were purified by 2% agarose gel electrophoresis using QIAquick gel kit. The PCR amplicons were immobilized on Streptavidin Sepharose HP beads (GE Healthcare Life Sciences) and subjected to pyrosequencing using the PyroMark MD System (QIAGEN) following manufacturer’s instructions. The methylation status of each CpG site was determined individually as an artificial C/T SNP using the QCpG software (QIAGEN). Bisulfite conversion efficiency was estimated based on the ratio of peak heights: (converted cytosines = T SNP) / ((converted cytosines = T SNP) + (unconverted cytosines = C SNP)). We used average value of two non-CpG sites closest to the sequencing primers. A series of methylated mtDNA controls were prepared by mixing NCAs fully methylated using the M.SssI CpG Methyltransferase (New England Biolabs) with unmethylated NCAs.

### Targeted bisulfite deep sequencing

PCR products were purified using QIAquick PCR purification kit (QIAGEN). Ion Torrent deep sequencing libraries were constructed from bisulfite-converted DNA using the KAPA Library Preparation Kit (Kapa Biosystems), quantified using the QIAxcel Advanced System (QIAGEN), and templated using the Ion PGM Template OT2 200 kit (Thermo Fisher). The libraries were sequenced using an Ion PGM Sequencing HiQ Kit with Ion 316 v2 Chips on the Ion Torrent PGM (Thermo Fisher), which generated non-directional ~200 nt length reads, 1500–7500 reads per library in the fastq format. Reads were aligned to human or mouse mtDNA reference sequences (hg38 and mm10, respectively) using the Bismark aligner [25] with bowtie2 option. Bisulfite conversion efficiency was evaluated based on cytosine conversion rate (C versus T) at non-CpG sites determined after visualization of the aligned reads (bam format) using the Integrative Genomics Viewer (IGV) [18].

### Shotgun bisulfite sequencing

Human mtDNA and nuclear DNA was isolated from 45 million human iPS cells (clone A4) using Mitochondrial DNA Isolation Kit (abcam) following the manufacturer’s instruction. Purity of human mtDNA was evaluated by qPCR detection of mtDNA markers (MT-ND1 and MT-ND5) and nuclear DNA markers (SLCO2B1 and SERPINA1) using Human mtDNA Monitoring Primer Set (Takara Bio). Purified mtDNA was shared to a target size of 200 bp using a Covaris S2 sonicator. Fragment size distribution was confirmed using Agilent High Sensitivity D1000 Screen Tape. Unmethylated lambda DNA isolated from infected GM119 strain of *E*. *coli* lacking both the *dam* and *dcm* DNA methyltransferases (Promega) was sheared separately to target the same size (200 bp). Equimolar mixture of shared mtDNA and lambda DNA was subjected to construction of Illumina shotgun bisulfite-seq deep sequencing libraries using NEBNEXT Ultra II DNA Library Prep Kit for Illumina, NEBNext Multiplex Oligos for Illumina (Methylated Adaptor, Index Primers Set 1), and the bisulfite conversion-compatible EpiMark Hot Start Taq DNA Polymerase (New England Biolabs). Bisulfite conversion was performed using EpiTect Fast DNA Bisulfite Kit (Qiagen). Libraries were quantified with KAPA Library Quantification Kit for Illumina Platform (KAPA Biosystems) and subjected to NextSeq 500 deep sequencing (75 nt, single-read). The deep sequencing data were analyzed using Bismark aligner [25] with bowtie2 option.

### Statistical analysis

Two-tailed t-test was considered statistically significant for *p* values less than 0.05. Linear regression lines and Pearson’s correlation coefficients were calculated using Microsoft EXCEL.

### Ethics statement

The human iPS cells involved in the present study were generated in-house from commercially available, de-identified neonatal foreskin fibroblasts and so were exempt from IRB approval. The mouse liver tissues were collected from adult C57BL/6 mice euthanized by carbon dioxide asphyxiation performed in strict accordance with the recommendation in the Guide for the Care and Use of Laboratory Animals of the National Institutes of Health. The animal experiment protocol, which specifically included the method of euthanasia using a carbon dioxide charging devise provided by the institution for this purpose as well as tissue collection from cadavers, was approved by Institutional Animal Care and Use Committee (IACUC) at the Massachusetts General Hospital (protocol# 2013N000077).

## Supporting information

S1 Fig Long-range, high-fidelity PCR amplification of human and mouse mtDNA to generate the negative control amplicons (NCAs). Human and mouse circular mtDNA was subjected to long, high-fidelity PCR amplification using the indicated primers and thermal cycler programs. The amplicons were subjected to 0.7% agarose gel electrophoresis to confirm their collect sizes (9.1 kb and 11.2 kb for human, 8 kb and 8.6 kb for mouse). M: Size markers.
(TIF)

S2 Fig Linearity of bisulfite pyrosequencing determination of CpG methylation in mixtures of methylated and unmethylated NCAs. Mixtures of unmethylated and *in vitro* methylated NCAs were subjected to bisulfite pyrosequencing with or without agarose gel purification prior to sequencing reactions. The regression curves were drawn using templates without gel purification. R^2^, square of Pearson’s correlation coefficient.
(TIF)

S3 Fig PCR and sequencing primers of bisulfite pyrosequencing and targeted bisulfite deep sequencing. (TIF)

S4 Fig Pyrograms of bisulfite pyrosequencing of NCAs containing 50% or 100% non-converted templates. The pyrograms were generated using the converted-template selective A9515 primer. Note significantly strong noise and very weak signals of the 100% unconverted template (bottom track).
(TIFF)

S5 Fig Bisulfite pyrosequencing of a non-CpG site in NCA mixtures with or without bisulfite conversion. A cytosine at a CpT site between CpGs #3 and 4 shown in Fig 4 was subjected to bisulfite pyrosequencing determination of bisulfite resistance. Each bar represents mean±SD of three independent assays.
(TIF)

S6 Fig Bisulfite pyrosequencing of ND1 gene methylation in intact and linearized human mtDNA. Bisulfite pyrosequencing of intact and circular mtDNA failed to generate specific signals and indistinguishable from the no-template control (NTC).
(TIFF)

S7 Fig **Pyrograms of ND1 gene in linearized, purified human mtDNA generated by sequencing primers hND1 (A) and A9515 (B).** Pyrograms generated by three independent assays are superimposed. NTC, no-template control.
(TIFF)

S8 Fig Bisulfite pyrosequencing of CYTB and ND1 genes in linearized, purified mouse mtDNA. Table shows percentages of methylation at CpG sites in mouse CYTB or ND1 gene. Pyrograms show raw data of bisulfite sequencing analysis of mouse CYTB and ND1 genes.
(TIFF)

S9 Fig Shotgun bisulfite sequencing of human iPSC mtDNA and unmethylated lambda DNA: Visualization by IGV. Cytosines marked with blue and red tags are converted to thymidines by bisulfite or not converted, respectively. (A), Unmethylated lambda DNA. (B) Human iPSC mtDNA, full-length. Locations of mtDNA-encoded genes and D-loop are indicated below. Genes encoding tRNAs are indicated with vertical lines without gene names. (C, D), ND1 (C) and CYTB (D) genes encoded in human iPSC mtDNA.
(TIF)

S10 Fig Shotgun sequencing of human iPSC mtDNA and unmethylated lambda DNA without bisulfite conversion. In the absence of bisulfite conversion, cytosines detected by deep sequencing are interpreted as methylated cytosines by software tools designed for bisulfite sequencing data analysis. (A, B) Cytosine methylation of mtDNA (A) and unmethylated lambda DNA (B). Percentage of cytosine methylation in the CpG and non-CpG contexts is shown with red and blue dots, respectively. Deep sequencing read coverage at cytosines is shown with green dots. In panel (A), locations of mtDNA-encoded genes and D-loop are indicated at the top, where positions of tRNA genes are shown with vertical bars without gene names.
(TIF)

S1 Table Targeted bisulfite deep sequencing of human (A) and mouse (B) mtDNA and NCAs.
(TIF)

S2 Table Shotgun bisulfite deep sequencing data for all CpG sites in human iPSC mtDNA and randomly selected CpG sites in unmethylated lambda DNA. (PDF)
