# Supplementary figures and images for: Technical adequacy of bisulfite sequencing and pyrosequencing for detection of mitochondrial DNA methylation: Sources and avoidance of false-positive detection
Source: PLoS One. 2018 Feb 8;13(2):e0192722. doi: 10.1371/journal.pone.0192722 (PMC5805350; doi:10.1371/journal.pone.0192722)

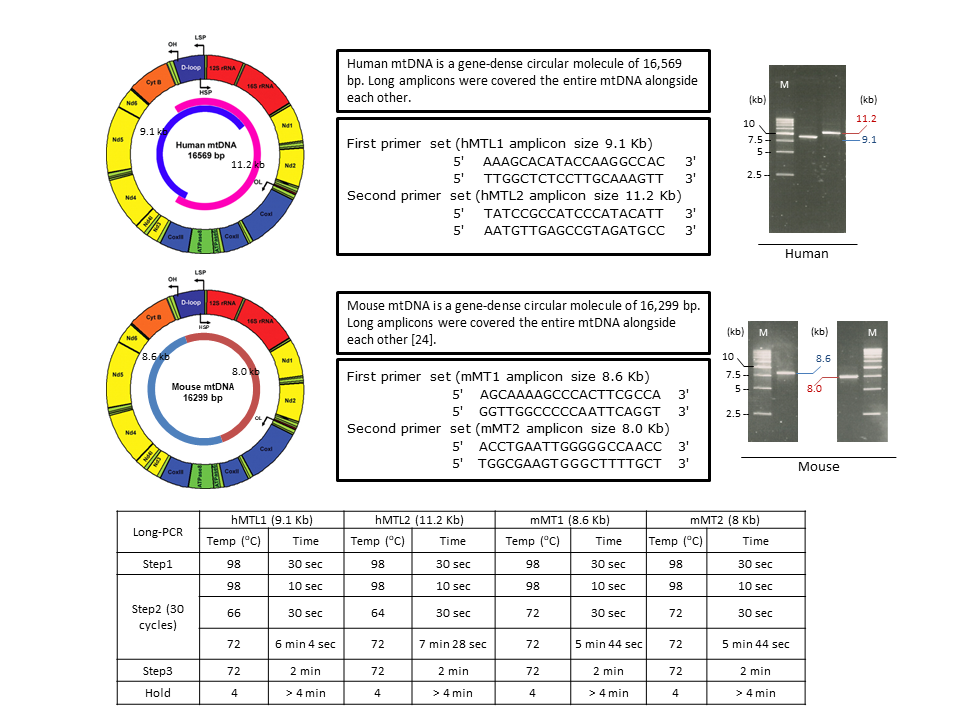

Supplement: S1 Fig — Human and mouse circular mtDNA was subjected to long, high-fidelity PCR amplification using the indicated primers and thermal cycler programs. The amplicons were subjected to 0.7% agarose gel electrophoresis to confirm their collect sizes (9.1 kb and 11.2 kb for human, 8 kb and 8.6 kb for mouse). M: Size markers. (TIF) [file pone.0192722.s001.tif]

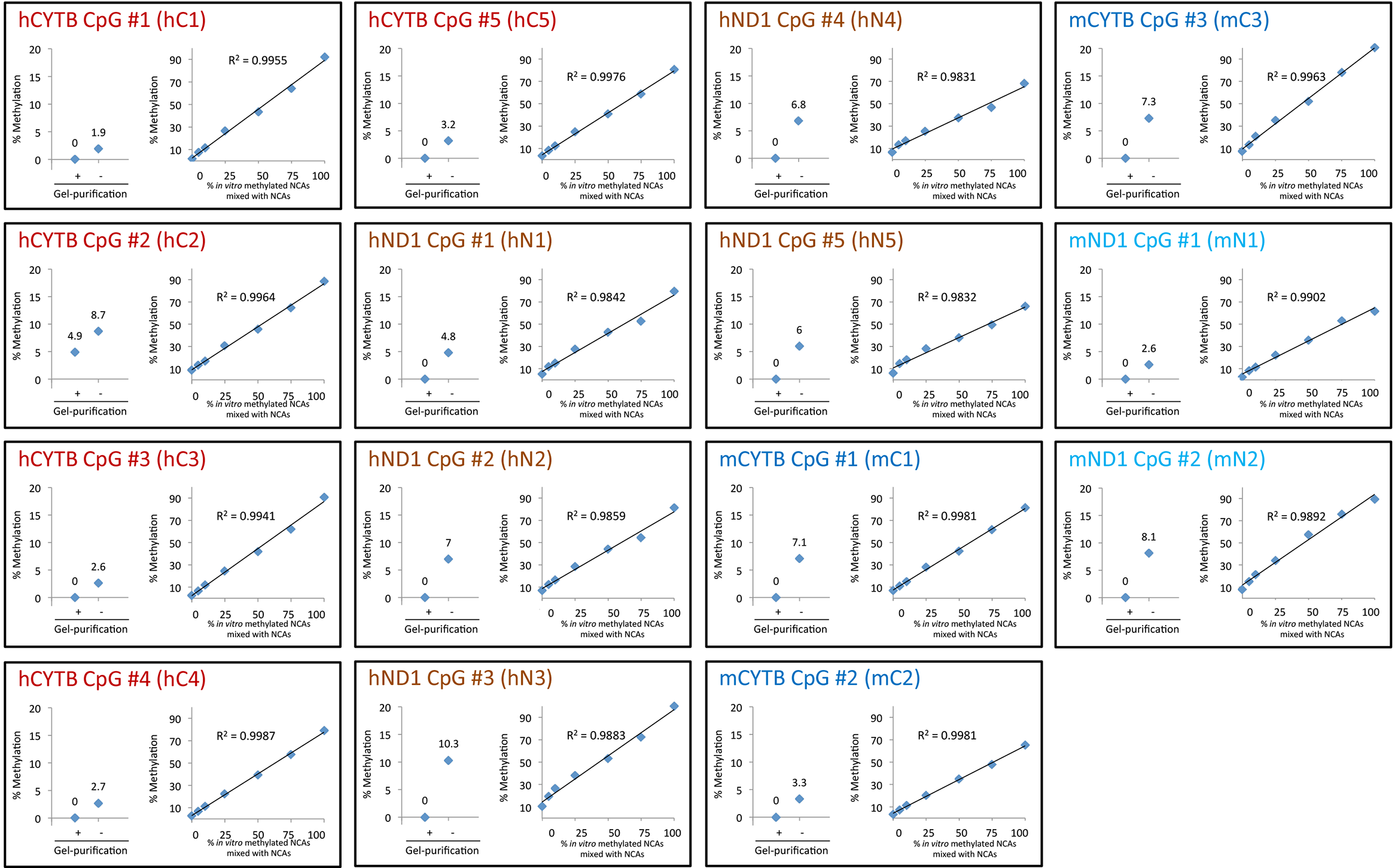

Supplement: S2 Fig — Mixtures of unmethylated and in vitro methylated NCAs were subjected to bisulfite pyrosequencing with or without agarose gel purification prior to sequencing reactions. The regression curves were drawn using templates without gel purification. R2, square of Pearson’s correlation coefficient. (TIF) [file pone.0192722.s002.tif]

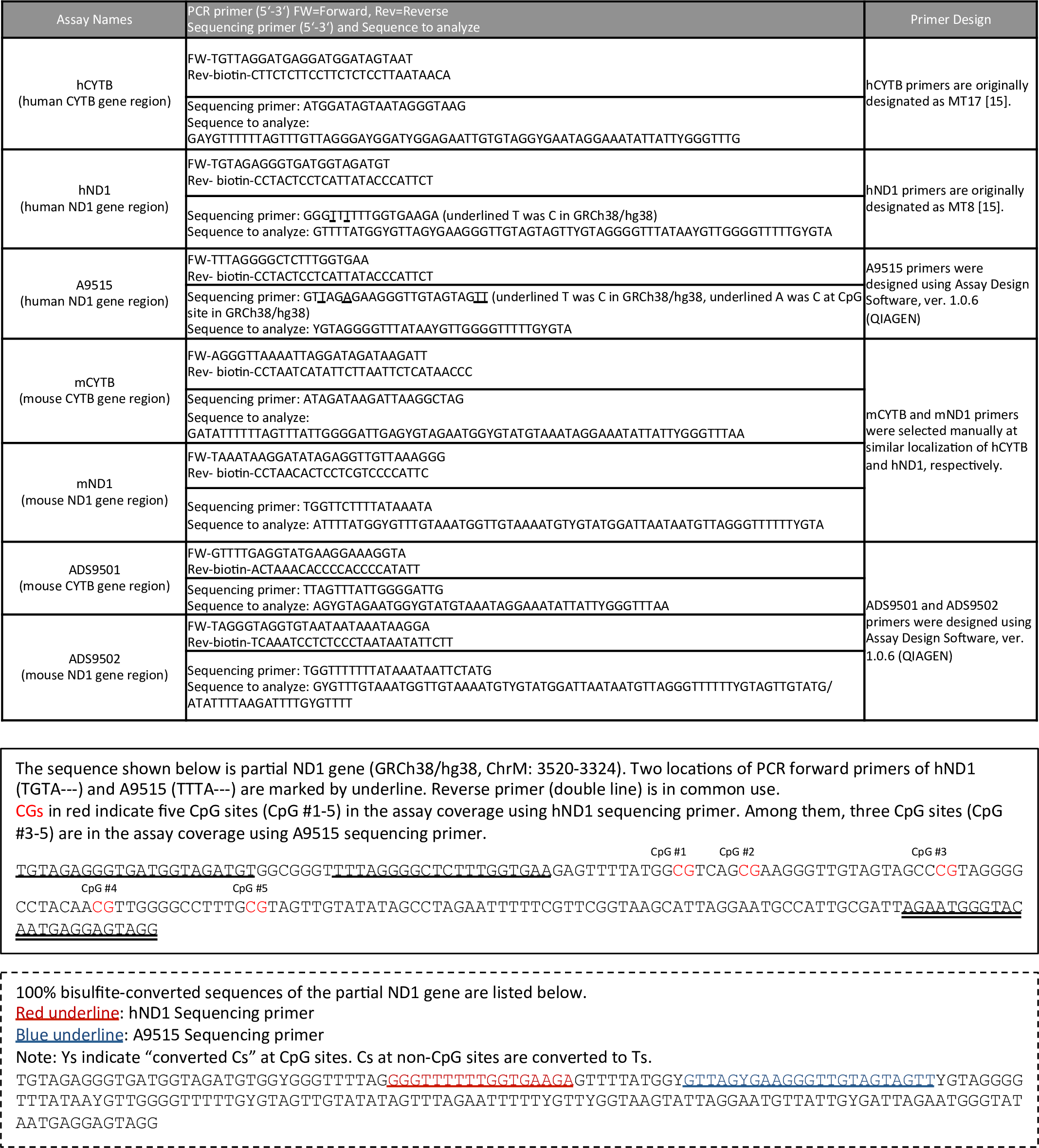

Supplement: S3 Fig — (TIF) [file pone.0192722.s003.tif]

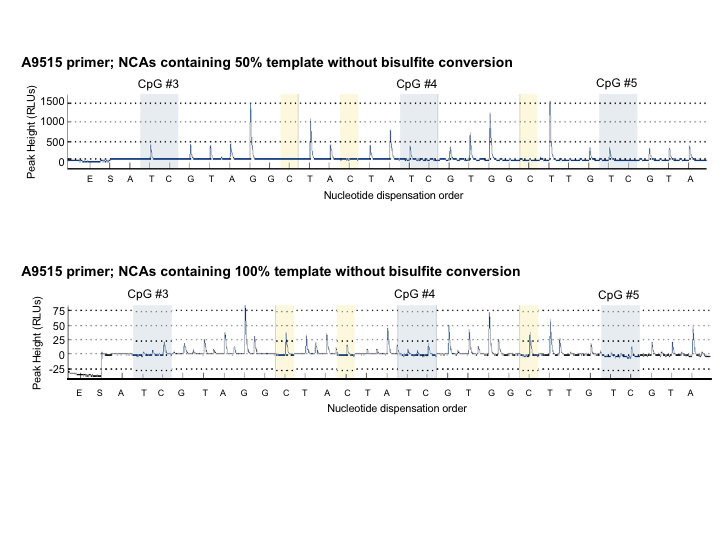

Supplement: S4 Fig — The pyrograms were generated using the converted-template selective A9515 primer. Note significantly strong noise and very weak signals of the 100% unconverted template (bottom track). (TIFF) [file pone.0192722.s004.tiff]

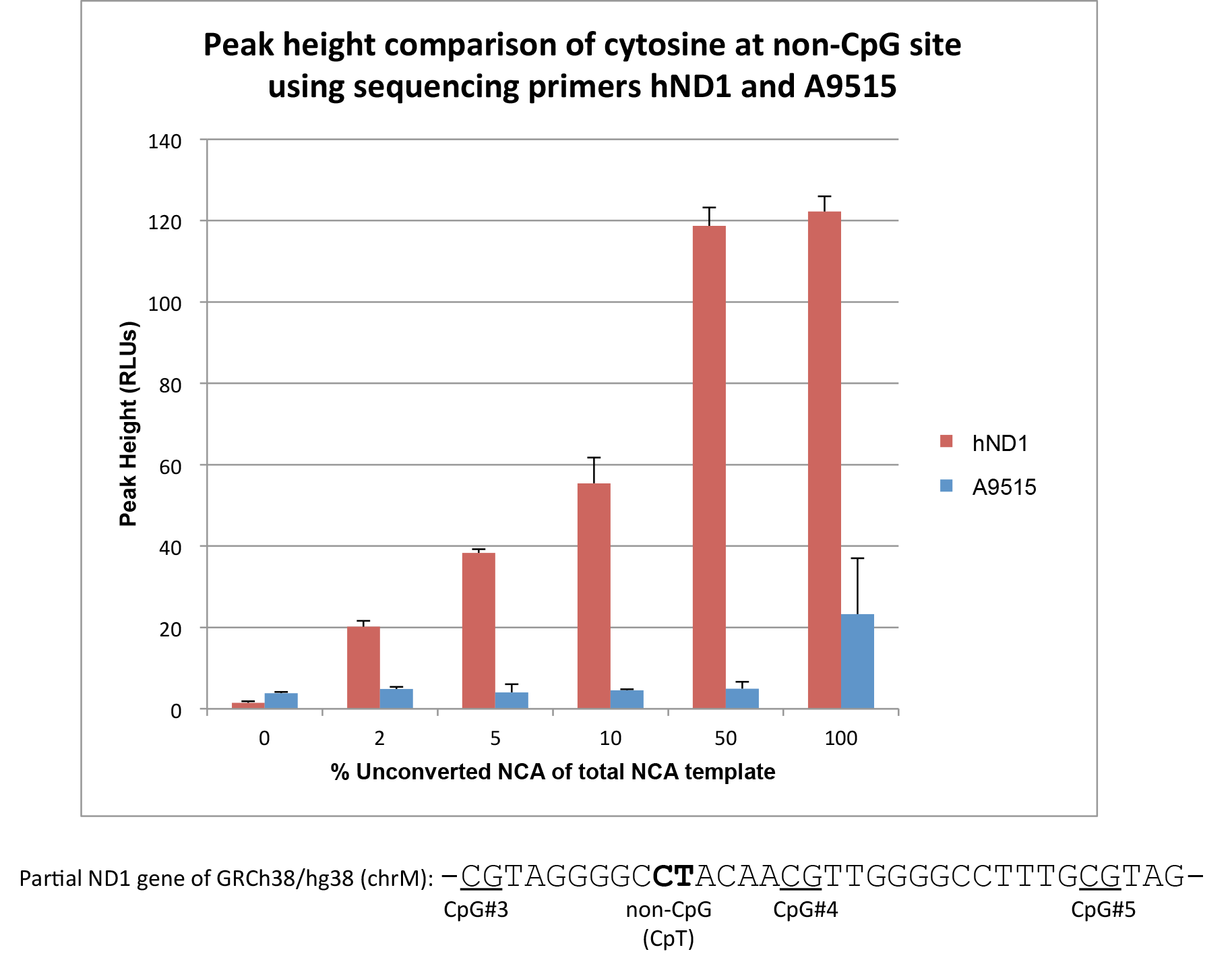

Supplement: S5 Fig — A cytosine at a CpT site between CpGs #3 and 4 shown in Fig 4 was subjected to bisulfite pyrosequencing determination of bisulfite resistance. Each bar represents mean±SD of three independent assays. (TIF) [file pone.0192722.s005.tif]

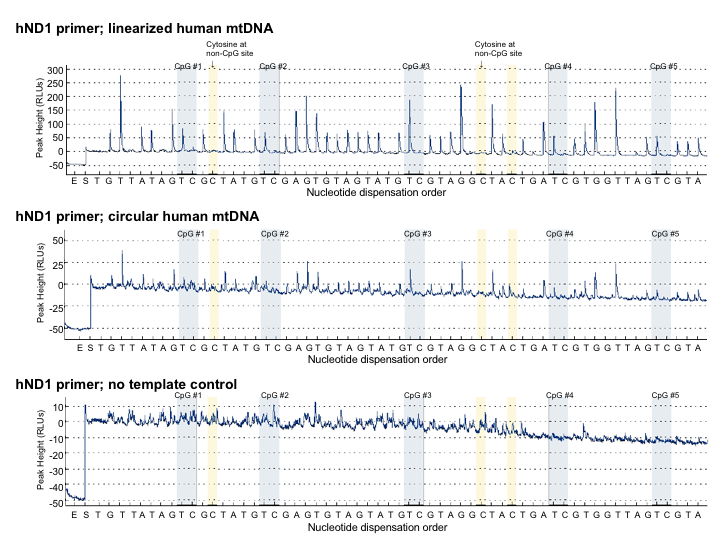

Supplement: S6 Fig — Bisulfite pyrosequencing of intact and circular mtDNA failed to generate specific signals and indistinguishable from the no-template control (NTC). (TIFF) [file pone.0192722.s006.tiff]

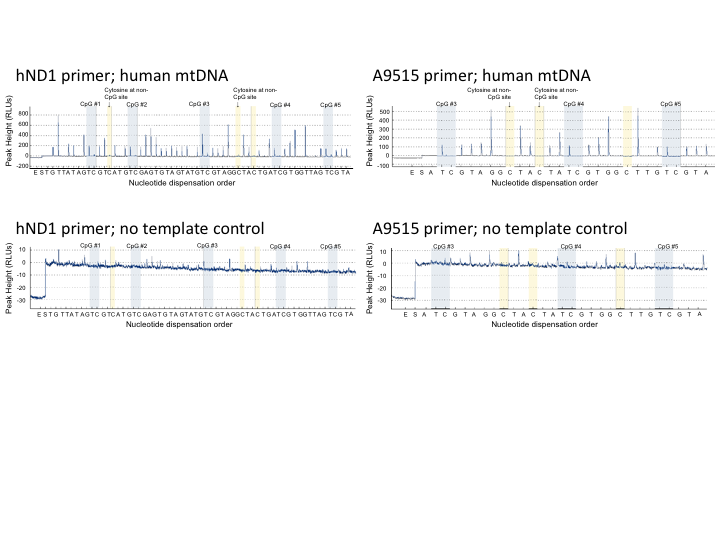

Supplement: S7 Fig — Pyrograms of ND1 gene in linearized, purified human mtDNA generated by sequencing primers hND1 (A) and A9515 (B). Pyrograms generated by three independent assays are superimposed. NTC, no-template control. (TIFF) [file pone.0192722.s007.tiff]

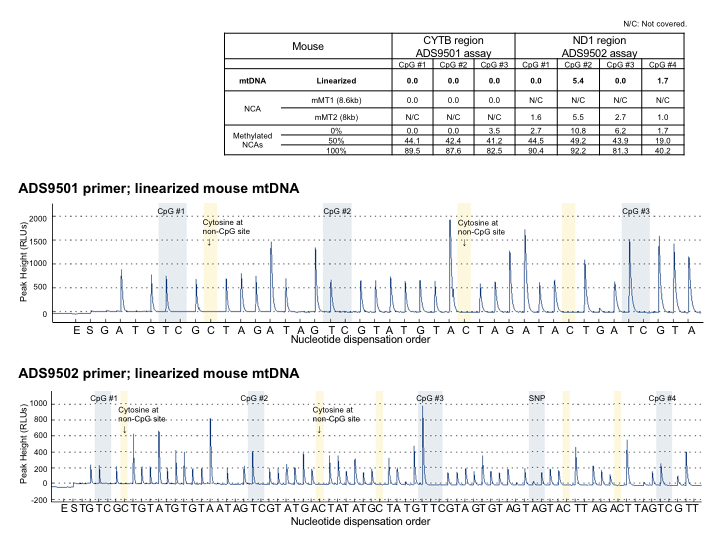

Supplement: S8 Fig — Table shows percentages of methylation at CpG sites in mouse CYTB or ND1 gene. Pyrograms show raw data of bisulfite sequencing analysis of mouse CYTB and ND1 genes. (TIFF) [file pone.0192722.s008.tiff]

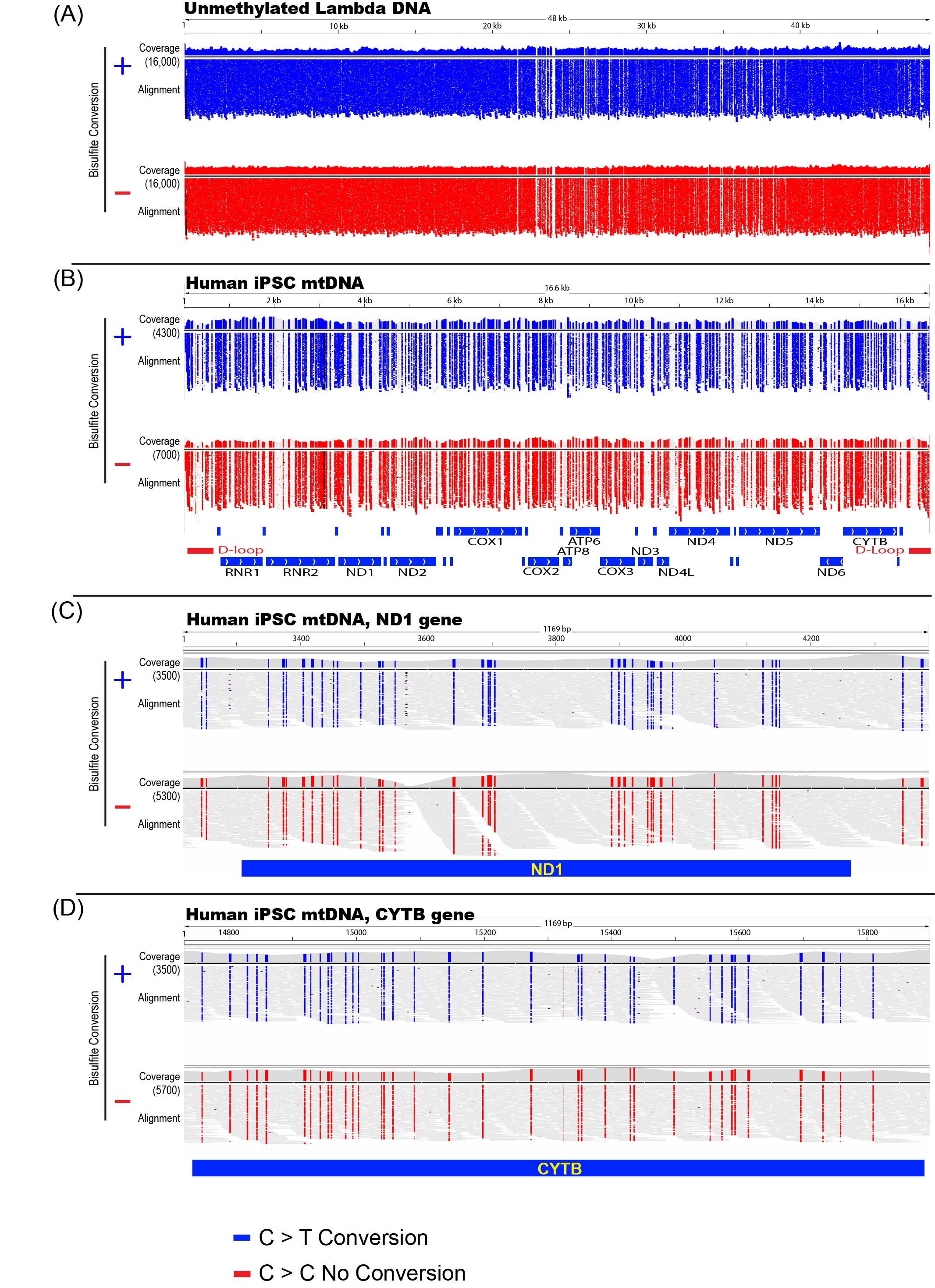

Supplement: S9 Fig — Cytosines marked with blue and red tags are converted to thymidines by bisulfite or not converted, respectively. (A), Unmethylated lambda DNA. (B) Human iPSC mtDNA, full-length. Locations of mtDNA-encoded genes and D-loop are indicated below. Genes encoding tRNAs are indicated with vertical lines without gene names. (C, D), ND1 (C) and CYTB (D) genes encoded in human iPSC mtDNA. (TIF) [file pone.0192722.s009.tif]

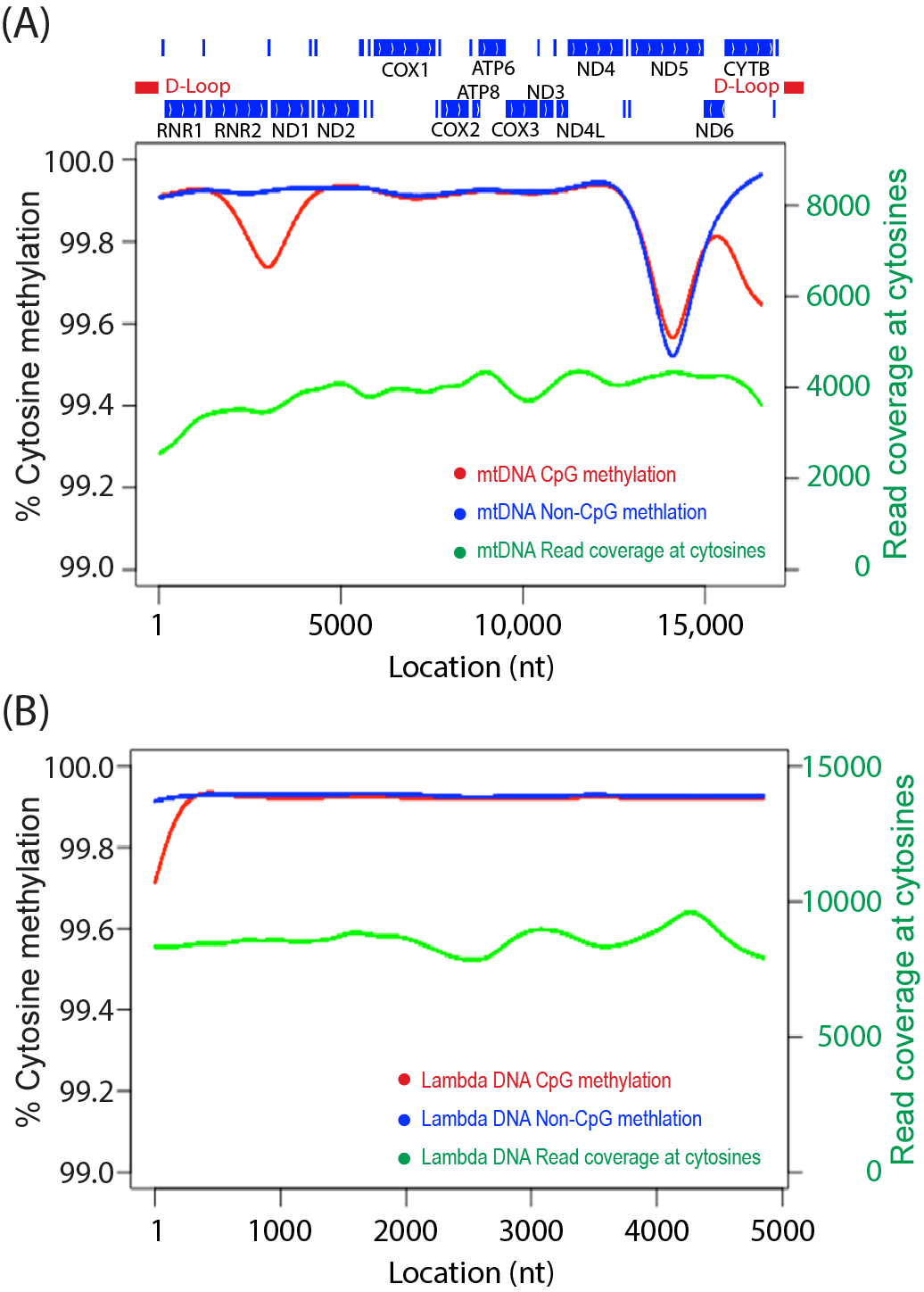

Supplement: S10 Fig — In the absence of bisulfite conversion, cytosines detected by deep sequencing are interpreted as methylated cytosines by software tools designed for bisulfite sequencing data analysis. (A, B) Cytosine methylation of mtDNA (A) and unmethylated lambda DNA (B). Percentage of cytosine methylation in the CpG and non-CpG contexts is shown with red and blue dots, respectively. Deep sequencing read coverage at cytosines is shown with green dots. In panel (A), locations of mtDNA-encoded genes and D-loop are indicated at the top, where positions of tRNA genes are shown with vertical bars without gene names. (TIF) [file pone.0192722.s010.tif]

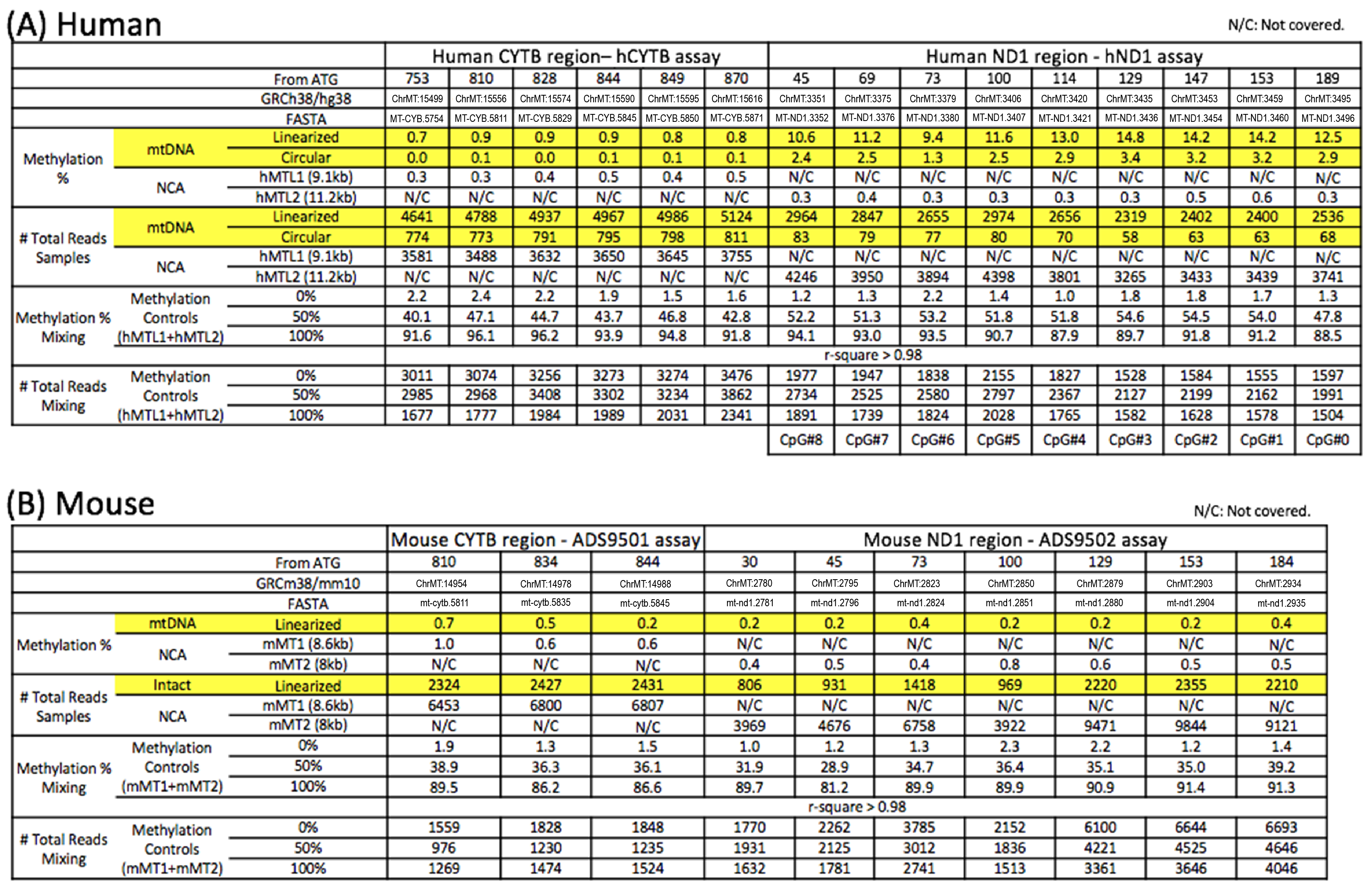

Supplement: S1 Table — Targeted bisulfite deep sequencing of human (A) and mouse (B) mtDNA and NCAs. (TIF) [file pone.0192722.s011.tif]
