# Supplementary material for: Technical adequacy of bisulfite sequencing and pyrosequencing for detection of mitochondrial DNA methylation: Sources and avoidance of false-positive detection
Source: PLoS One. 2018 Feb 8;13(2):e0192722. doi: 10.1371/journal.pone.0192722 (PMC5805350; doi:10.1371/journal.pone.0192722)
Supplement: S2 Table — (PDF) [file pone.0192722.s012.pdf]

Human iPSC mtDNA	Domain	Gene	Product	Location	A call	C call	G call	T call	Total	% CpG methylated	% CpG unmethylated	% Seq Error	% Methyl Mean	% Methyl SD
	tRNA	TRNF	tRNA-Phe	624	1	15	0	2669	2685	0.559	99.441	0.037	0.559	
	rRNA	RNR1	s-rRNA	708	2	8	0	2758	2768	0.289	99.711	0.072	0.651	0.181
				739	0	13	0	2619	2632	0.494	99.506	0.000		
				765	0	14	0	2680	2694	0.520	99.480	0.000		
				785	0	16	0	2661	2677	0.598	99.402	0.000		
				808	1	10	0	2675	2686	0.372	99.628	0.037		
				842	0	19	0	2933	2952	0.644	99.356	0.000		
				886	1	19	0	3140	3160	0.601	99.399	0.032		
				898	1	27	0	2948	2976	0.908	99.092	0.034		
				900	3	21	0	2894	2918	0.720	99.280	0.103		
				908	1	27	0	2872	2900	0.931	99.069	0.034		
				932	2	18	1	2511	2532	0.712	99.288	0.118		
				935	3	20	0	2464	2487	0.805	99.195	0.121		
				1023	1	25	0	3164	3190	0.784	99.216	0.031		
				1133	3	16	1	2631	2651	0.604	99.396	0.151		
				1146	5	23	2	2692	2722	0.847	99.153	0.257		
				1177	2	21	1	2495	2519	0.835	99.165	0.119		
				1216	3	19	0	2344	2366	0.804	99.196	0.127		
				1226	3	7	0	2364	2374	0.295	99.705	0.126		
				1262	1	18	1	2798	2818	0.639	99.361	0.071		
				1302	1	23	0	3490	3514	0.655	99.345	0.028		
				1314	0	31	0	3648	3679	0.843	99.157	0.000		
				1322	0	21	2	3815	3838	0.547	99.453	0.052		
				1388	2	26	1	3649	3678	0.707	99.293	0.082		
				1414	0	34	1	3561	3596	0.946	99.054	0.028		
				1473	3	11	0	2916	2930	0.376	99.624	0.102		
				1475	1	21	0	2903	2925	0.718	99.282	0.034		
				1484	1	18	0	2733	2752	0.654	99.346	0.036		
				1488	0	11	0	2716	2727	0.403	99.597	0.000		
				1537	1	24	1	3124	3150	0.762	99.238	0.063		
				1561	3	20	0	3727	3750	0.534	99.466	0.080		
				1597	2	22	0	3380	3404	0.647	99.353	0.059		
	tRNA	TRNV	tRNA-Val	1663	2	6	0	2139	2147	0.280	99.720	0.093	0.280	
	rRNA	RNR2	l-rRNA	1749	1	30	1	3448	3480	0.863	99.137	0.057	0.680	0.168
				1769	2	34	1	3920	3957	0.860	99.140	0.076		
				1786	4	26	0	4102	4132	0.630	99.370	0.097		
				1905	1	28	1	3971	4001	0.700	99.300	0.050		
				1915	3	27	1	4092	4123	0.655	99.345	0.097		
				1948	1	28	0	3888	3917	0.715	99.285	0.026		
				1989	0	35	0	3897	3932	0.890	99.110	0.000		
				2001	4	31	1	3805	3841	0.808	99.192	0.130		
				2202	1	27	0	2683	2711	0.996	99.004	0.037		
				2332	1	11	0	2846	2858	0.385	99.615	0.035		
				2344	1	17	1	2878	2897	0.587	99.413	0.069		
				2476	2	34	0	3426	3462	0.983	99.017	0.058		
				2491	4	30	0	3286	3320	0.905	99.095	0.120		
				2541	6	20	0	2677	2703	0.742	99.258	0.222		
				2566	0	21	4	2752	2777	0.757	99.243	0.144		
				2570	2	17	0	2763	2782	0.612	99.388	0.072		
				2572	5	25	1	2778	2809	0.892	99.108	0.214		
				2584	0	28	0	2884	2912	0.962	99.038	0.000		
				2642	3	23	1	3379	3406	0.676	99.324	0.117		
				2689	1	20	0	3260	3281	0.610	99.390	0.030		
				2699	4	18	0	3158	3180	0.567	99.433	0.126		
				2718	0	18	0	2967	2985	0.603	99.397	0.000		
				2809	1	15	0	2401	2417	0.621	99.379	0.041		
				2818	1	11	1	2382	2395	0.460	99.540	0.084		
				2824	0	10	0	2411	2421	0.413	99.587	0.000		
				2843	0	14	0	2329	2343	0.598	99.402	0.000		
				2877	4	12	1	2600	2617	0.459	99.541	0.191		
				2915	1	18	0	3054	3073	0.586	99.414	0.033		
				2942	3	28	1	3231	3263	0.859	99.141	0.123		
				2982	1	21	1	3279	3302	0.636	99.364	0.061		
				2988	2	24	0	3297	3323	0.723	99.277	0.060		
				3021	1	25	0	3198	3224	0.776	99.224	0.031		
				3035	0	18	0	3288	3306	0.544	99.456	0.000		
				3046	0	27	2	3254	3283	0.823	99.177	0.061		
				3060	1	22	0	3241	3264	0.674	99.326	0.031		
				3078	0	23	0	3015	3038	0.757	99.243	0.000		
				3093	3	16	0	2915	2934	0.546	99.454	0.102		
				3126	3	11	0	2580	2594	0.425	99.575	0.116		
				3162	0	14	0	2548	2562	0.546	99.454	0.000		
				3172	2	9	3	2404	2418	0.373	99.627	0.207		
	tRNA	TRNL1	tRNA-Leu	3247	2	14	0	2584	2600	0.539	99.461	0.077	0.582	0.061
				3254	3	16	0	2542	2561	0.625	99.375	0.117		
	protein	ND1	NADH dehydrogenase subunit 1	3351	0	7	1	2171	2179	0.321	99.679	0.046	0.522	0.183
				3375	1	12	0	2333	2346	0.512	99.488	0.043		
				3379	4	23	0	2277	2304	1.000	99.000	0.174		
				3406	1	10	0	2304	2315	0.432	99.568	0.043		
				3420	0	11	0	2259	2270	0.485	99.515	0.000		
				3435	0	11	0	2023	2034	0.541	99.459	0.000		
				3453	0	13	0	1796	1809	0.719	99.281	0.000		
				3459	0	12	1	1717	1730	0.694	99.306	0.058		
				3495	1	7	0	1499	1507	0.465	99.535	0.066		
				3525	0	12	0	1700	1712	0.701	99.299	0.000		
				3530	1	3	0	1729	1733	0.173	99.827	0.058		
				3549	0	14	0	1770	1784	0.785	99.215	0.000		

				3642	1	14	0	2189	2204	0.635	99.365	0.045		
				3687	0	8	0	2104	2112	0.379	99.621	0.000		
				3696	0	10	1	2061	2072	0.483	99.517	0.048		
				3699	1	10	1	2040	2052	0.488	99.512	0.097		
				3706	4	6	0	1988	1998	0.301	99.699	0.200		
				3889	0	8	1	2336	2345	0.341	99.659	0.043		
				3900	2	13	1	2257	2273	0.573	99.427	0.132		
				3909	0	7	1	2293	2301	0.304	99.696	0.043		
				3921	0	6	0	2282	2288	0.262	99.738	0.000		
				3945	2	12	0	1964	1978	0.607	99.393	0.101		
				3951	0	6	0	1885	1891	0.317	99.683	0.000		
				3954	0	12	0	1795	1807	0.664	99.336	0.000		
				3966	0	9	0	1637	1646	0.547	99.453	0.000		
				3984	0	10	1	1495	1506	0.664	99.336	0.066		
				4050	1	11	1	2132	2145	0.513	99.487	0.093		
				4126	0	16	0	2053	2069	0.773	99.227	0.000		
				4141	1	7	0	2088	2096	0.334	99.666	0.048		
				4147	2	12	0	2040	2054	0.585	99.415	0.097		
				4152	1	12	1	2036	2050	0.586	99.414	0.098		
	tRNA	TRNQ	tRNA-Gln	4345	1	23	0	3019	3043	0.756	99.244	0.033	0.592	0.231
				4375	3	10	0	2322	2335	0.429	99.571	0.128		
	tRNA	TRNM	tRNA-Met	4426	4	12	0	2342	2358	0.510	99.490	0.170	0.517	0.109
				4439	3	15	0	2365	2383	0.630	99.370	0.126		
				4463	0	10	2	2419	2431	0.412	99.588	0.082		
	protein	ND2	NADH dehydrogenase subunit 2	4490	2	12	0	2354	2368	0.507	99.493	0.084	0.603	0.161
				4531	1	16	0	2420	2437	0.657	99.343	0.041		
				4540	0	13	1	2463	2477	0.525	99.475	0.040		
				4620	0	16	0	2575	2591	0.618	99.382	0.000		
				4654	3	18	2	2165	2188	0.825	99.175	0.229		
				4664	3	7	0	2046	2056	0.341	99.659	0.146		
				4712	1	10	0	1622	1633	0.613	99.387	0.061		
				4847	0	14	0	2101	2115	0.662	99.338	0.000		
				4919	1	21	0	2068	2090	1.005	98.995	0.048		
				4995	1	14	0	2274	2289	0.612	99.388	0.044		
				5053	1	10	1	1844	1856	0.539	99.461	0.108		
				5111	1	9	0	1874	1884	0.478	99.522	0.053		
				5146	0	9	0	2076	2085	0.432	99.568	0.000		
				5163	0	17	0	2031	2048	0.830	99.170	0.000		
				5236	0	8	0	1719	1727	0.463	99.537	0.000		
				5243	1	12	0	1769	1782	0.674	99.326	0.056		
				5270	0	9	0	1731	1740	0.517	99.483	0.000		
				5352	0	8	0	1561	1569	0.510	99.490	0.000		
				5399	0	15	0	1892	1907	0.787	99.213	0.000		
				5459	1	14	0	2110	2125	0.659	99.341	0.047		
				5470	1	8	0	1929	1938	0.413	99.587	0.052		
	tRNA	TRNA	tRNA-Ala	5622	2	23	0	2645	2670	0.862	99.138	0.075	0.643	0.166
	OL			5737	1	13	0	2503	2517	0.517	99.483	0.040		
				5740	1	13	0	2483	2497	0.521	99.479	0.040		
				5743	1	20	1	2537	2559	0.782	99.218	0.078		
				5755	5	15	1	2791	2812	0.535	99.465	0.213		
	tRNA	TRNC	tRNA-Cys	5767	2	17	0	2854	2873	0.592	99.408	0.070	0.556	0.079
				5790	1	14	0	2992	3007	0.466	99.534	0.033		
				5820	2	19	1	3097	3119	0.610	99.390	0.096		
	protein	COX1	cytochrome oxidase subunit 1	5909	4	11	0	2229	2244	0.491	99.509	0.178	0.642	0.198
				5912	2	19	0	2278	2299	0.827	99.173	0.087		
				5916	2	6	1	2318	2327	0.258	99.742	0.129		
				5969	0	11	0	2442	2453	0.448	99.552	0.000		
				5972	2	14	1	2439	2456	0.571	99.429	0.122		
				6015	1	18	0	2387	2406	0.748	99.252	0.042		
				6020	2	14	1	2436	2453	0.571	99.429	0.122		
				6053	1	17	0	2394	2412	0.705	99.295	0.041		
				6068	0	15	0	2516	2531	0.593	99.407	0.000		
				6074	2	16	0	2549	2567	0.624	99.376	0.078		
				6128	4	28	1	2918	2951	0.950	99.050	0.169		
				6164	4	21	0	2742	2767	0.760	99.240	0.145		
				6173	0	19	0	2582	2601	0.730	99.270	0.000		
				6181	3	26	1	2534	2564	1.016	98.984	0.156		
				6189	1	16	0	2321	2338	0.685	99.315	0.043		
				6242	2	20	2	1930	1954	1.026	98.974	0.205		
				6263	0	12	2	2177	2191	0.548	99.452	0.091		
				6329	1	9	1	2077	2088	0.431	99.569	0.096		
				6445	1	11	1	2266	2279	0.483	99.517	0.088		
				6455	0	7	0	2243	2250	0.311	99.689	0.000		
				6464	1	7	0	2250	2258	0.310	99.690	0.044		
				6540	2	13	0	1832	1847	0.705	99.295	0.108		
				6563	0	16	0	1870	1886	0.848	99.152	0.000		
				6569	1	8	2	1899	1910	0.420	99.580	0.157		
				6572	0	10	0	1950	1960	0.510	99.490	0.000		
				6617	2	22	1	2681	2706	0.814	99.186	0.111		
				6656	3	16	1	3187	3207	0.500	99.500	0.125		
				6689	0	15	0	3550	3565	0.421	99.579	0.000		
				6761	1	24	0	3132	3157	0.760	99.240	0.032		
				6797	2	19	0	2850	2871	0.662	99.338	0.070		
				6807	1	20	0	2867	2888	0.693	99.307	0.035		
				6824	0	20	0	2829	2849	0.702	99.298	0.000		
				6839	0	17	0	2854	2871	0.592	99.408	0.000		
				6851	2	13	0	2854	2869	0.453	99.547	0.070		
				6854	2	18	1	2879	2900	0.621	99.379	0.103		

				6875	0	17	0	2998	3015	0.564	99.436	0.000		
				6887	1	32	0	3063	3096	1.034	98.966	0.032		
				6950	2	18	2	2868	2890	0.624	99.376	0.138		
				6998	1	24	0	2830	2855	0.841	99.159	0.035		
				7007	1	17	0	2768	2786	0.610	99.390	0.036		
				7012	1	20	0	2809	2830	0.707	99.293	0.035		
				7019	1	19	0	2871	2891	0.657	99.343	0.035		
				7130	2	9	0	2110	2121	0.425	99.575	0.094		
				7160	2	16	0	2127	2145	0.747	99.253	0.093		
				7163	2	19	0	2135	2156	0.882	99.118	0.093		
				7196	0	14	2	2287	2303	0.608	99.392	0.087		
				7205	2	9	0	2437	2448	0.368	99.632	0.082		
				7215	1	18	0	2459	2478	0.727	99.273	0.040		
				7218	1	18	1	2497	2517	0.716	99.284	0.079		
				7225	2	22	1	2503	2528	0.871	99.129	0.119		
				7235	0	28	0	2528	2556	1.095	98.905	0.000		
				7331	0	19	0	3432	3451	0.551	99.449	0.000		
				7336	1	25	1	3401	3428	0.730	99.270	0.058		
				7341	2	8	2	3433	3445	0.232	99.768	0.116		
				7418	1	19	1	2490	2511	0.757	99.243	0.080		
				7427	1	12	0	2768	2781	0.432	99.568	0.036		
	tRNA	TRNS1	tRNA-Ser	7462	1	24	0	3576	3601	0.667	99.333	0.028	0.667	
	protein	COX2	cytochrome oxidase subunit 2	7599	5	27	0	3235	3267	0.828	99.172	0.153	0.637	0.180
				7618	2	20	1	3081	3104	0.645	99.355	0.097		
				7663	0	13	0	2464	2477	0.525	99.475	0.000		
				7756	1	9	1	2476	2487	0.362	99.638	0.080		
				7774	1	20	0	2579	2600	0.770	99.230	0.038		
				7792	1	21	0	2573	2595	0.810	99.190	0.039		
				7813	0	14	0	2329	2343	0.598	99.402	0.000		
				7829	2	25	1	1983	2011	1.245	98.755	0.149		
				7849	0	11	0	2065	2076	0.530	99.470	0.000		
				7858	0	12	1	2126	2139	0.561	99.439	0.047		
				7909	3	16	0	2371	2390	0.670	99.330	0.126		
				7918	1	8	0	2408	2417	0.331	99.669	0.041		
				7924	0	15	0	2374	2389	0.628	99.372	0.000		
				7927	0	20	0	2289	2309	0.866	99.134	0.000		
				7978	2	15	0	2559	2576	0.583	99.417	0.078		
				7985	1	21	0	2628	2650	0.793	99.207	0.038		
				7996	1	16	2	2629	2648	0.605	99.395	0.113		
				8006	2	19	0	2662	2683	0.709	99.291	0.075		
				8019	2	15	0	2660	2677	0.561	99.439	0.075		
				8036	3	19	0	2647	2669	0.713	99.287	0.112		
				8059	0	16	0	2670	2686	0.596	99.404	0.000		
				8113	0	10	1	2702	2713	0.369	99.631	0.037		
				8117	2	13	0	2663	2678	0.486	99.514	0.075		
				8140	2	13	0	2587	2602	0.500	99.500	0.077		
				8147	1	13	0	2641	2655	0.490	99.510	0.038		
				8151	0	17	0	2733	2750	0.618	99.382	0.000		
				8164	4	22	0	2813	2839	0.776	99.224	0.141		
				8212	0	19	0	2834	2853	0.666	99.334	0.000		
				8254	1	18	0	2808	2827	0.637	99.363	0.035		
	protein	ATP8	ATP synthase F0 subunit 8	8386	1	10	0	1971	1982	0.505	99.495	0.050	0.608	0.090
				8532	2	24	1	3640	3667	0.655	99.345	0.082		
				8544	4	23	1	3436	3464	0.665	99.335	0.144		
	protein	ATP6	ATP synthase F0 subunit 6	8580	1	13	0	2352	2366	0.550	99.450	0.042	0.641	0.178
				8583	1	14	0	2248	2263	0.619	99.381	0.044		
				8647	0	18	0	2041	2059	0.874	99.126	0.000		
				8722	0	10	0	2444	2454	0.407	99.593	0.000		
				8781	0	12	2	1866	1880	0.639	99.361	0.106		
				8855	2	9	2	1894	1907	0.473	99.527	0.210		
				8878	0	18	0	2129	2147	0.838	99.162	0.000		
				8958	0	8	0	1953	1961	0.408	99.592	0.000		
				8997	2	16	0	2191	2209	0.725	99.275	0.091		
				9001	1	18	2	2206	2227	0.809	99.191	0.135		
				9009	0	7	0	2177	2184	0.321	99.679	0.000		
				9054	0	17	0	1916	1933	0.879	99.121	0.000		
				9138	0	17	0	2472	2489	0.683	99.317	0.000		
				9144	0	18	0	2432	2450	0.735	99.265	0.000		
				9162	2	13	1	2458	2474	0.526	99.474	0.121		
				9195	0	17	0	2189	2206	0.771	99.229	0.000		
	protein	COX3	cytochrome oxidase subunit 3	9293	2	14	0	1853	1869	0.750	99.250	0.107	0.565	0.180
				9328	1	12	0	1615	1628	0.738	99.262	0.061		
				9381	2	18	1	2206	2227	0.809	99.191	0.135		
				9383	0	16	1	2275	2292	0.698	99.302	0.044		
				9393	2	21	1	2518	2542	0.827	99.173	0.118		
				9444	0	18	2	2748	2768	0.651	99.349	0.072		
				9449	2	20	0	2754	2776	0.721	99.279	0.072		
				9488	0	13	17	2501	2531	0.517	99.483	0.672		
				9574	0	7	1	2104	2112	0.332	99.668	0.047		
				9611	0	16	0	2497	2513	0.637	99.363	0.000		
				9620	0	21	0	2570	2591	0.810	99.190	0.000		
				9672	2	20	0	3123	3145	0.636	99.364	0.064		
				9752	1	10	1	2719	2731	0.366	99.634	0.073		
				9773	0	9	0	2426	2435	0.370	99.630	0.000		
				9776	1	13	0	2454	2468	0.527	99.473	0.041		
				9785	3	13	0	2494	2510	0.519	99.481	0.120		
				9818	0	10	1	2286	2297	0.436	99.564	0.044		
				9827	2	11	0	2263	2276	0.484	99.516	0.088		

				9867	0	10	0	2112	2122	0.471	99.529	0.000		
				9911	1	8	0	2866	2875	0.278	99.722	0.035		
				9917	1	19	0	3019	3039	0.625	99.375	0.033		
				9920	2	7	0	3071	3080	0.227	99.773	0.065		
	tRNA	TRNG	tRNA-Gly	10013	1	23	0	3197	3221	0.714	99.286	0.031	0.714	
	protein	ND3	NADH dehydrogenase subunit 3	10067	1	26	0	2830	2857	0.910	99.090	0.035	0.501	0.211
				10142	2	13	0	2541	2556	0.509	99.491	0.078		
				10169	1	16	2	2603	2622	0.611	99.389	0.114		
				10175	3	8	1	2689	2701	0.297	99.703	0.148		
				10181	1	17	0	2563	2581	0.659	99.341	0.039		
				10196	1	9	0	2466	2476	0.364	99.636	0.040		
				10200	2	11	2	2454	2469	0.446	99.554	0.162		
				10202	1	5	0	2448	2454	0.204	99.796	0.041		
				10400	2	19	1	3696	3718	0.511	99.489	0.081		
	tRNA	TRNR	tRNA-Arg	10426	1	23	1	3386	3411	0.675	99.325	0.059	0.586	0.125
				10436	2	16	0	3202	3220	0.497	99.503	0.062		
	protein	ND4L	NADH dehydrogenase subunit 4L	10536	1	12	0	2397	2410	0.498	99.502	0.041	0.528	0.178
				10585	1	10	0	2313	2324	0.430	99.570	0.043		
				10670	1	5	1	1888	1895	0.264	99.736	0.106		
				10676	0	12	1	1882	1895	0.634	99.366	0.053		
				10684	1	15	0	1894	1910	0.786	99.214	0.052		
				10736	0	11	0	1966	1977	0.556	99.444	0.000		
	protein	ND4	NADH dehydrogenase subunit 4	10774	3	15	0	2256	2274	0.661	99.339	0.132	0.609	0.179
				10933	4	7	0	1778	1789	0.392	99.608	0.224		
				11003	1	5	0	2137	2143	0.233	99.767	0.047		
				11030	1	14	0	2201	2216	0.632	99.368	0.045		
				11125	0	18	3	2096	2117	0.851	99.149	0.142		
				11162	0	20	1	2372	2393	0.836	99.164	0.042		
				11183	0	12	1	2227	2240	0.536	99.464	0.045		
				11191	0	17	0	2102	2119	0.802	99.198	0.000		
				11245	0	10	0	1528	1538	0.650	99.350	0.000		
				11389	3	14	0	3094	3111	0.450	99.550	0.096		
				11422	1	20	0	2763	2784	0.719	99.281	0.036		
				11434	0	6	0	2622	2628	0.228	99.772	0.000		
				11455	0	20	0	2334	2354	0.850	99.150	0.000		
				11476	0	14	0	2210	2224	0.629	99.371	0.000		
				11492	0	16	1	2270	2287	0.700	99.300	0.044		
				11591	3	19	1	2675	2698	0.705	99.295	0.148		
				11610	1	14	0	2528	2543	0.551	99.449	0.039		
				11647	3	14	0	2334	2351	0.596	99.404	0.128		
				11689	2	16	0	2124	2142	0.748	99.252	0.093		
				11692	2	16	0	2068	2086	0.768	99.232	0.096		
				11710	2	7	1	2063	2073	0.338	99.662	0.145		
				11716	1	7	0	2067	2075	0.338	99.662	0.048		
				11761	1	15	0	2368	2384	0.629	99.371	0.042		
				11765	0	15	0	2414	2429	0.618	99.382	0.000		
				11777	2	13	0	2397	2412	0.539	99.461	0.083		
				11851	1	7	0	2288	2296	0.305	99.695	0.044		
				11860	1	16	0	2145	2162	0.740	99.260	0.046		
				11913	2	13	0	1915	1930	0.674	99.326	0.104		
				12053	1	16	0	1987	2004	0.799	99.201	0.050		
				12112	0	16	0	2345	2361	0.678	99.322	0.000		
				12124	1	17	0	2443	2461	0.691	99.309	0.041		
	tRNA	TRNH	tRNA-His	12191	1	22	0	3382	3405	0.646	99.354	0.029	0.694	0.067
				12206	2	25	0	3347	3374	0.741	99.259	0.059		
	protein	ND5	NADH dehydrogenase subunit 5	12405	1	7	0	2000	2008	0.349	99.651	0.050	0.554	0.147
				12456	1	11	0	2398	2410	0.457	99.543	0.041		
				12527	1	14	0	2365	2380	0.588	99.412	0.042		
				12621	0	14	0	2431	2445	0.573	99.427	0.000		
				12735	1	12	1	2395	2409	0.499	99.501	0.083		
				12672	1	13	1	2604	2619	0.497	99.503	0.076		
				12774	2	16	1	2753	2772	0.578	99.422	0.108		
				12813	1	14	2	2911	2928	0.479	99.521	0.102		
				12817	0	17	0	2862	2879	0.590	99.410	0.000		
				12862	1	15	1	2385	2402	0.625	99.375	0.083		
				12867	0	17	0	2335	2352	0.723	99.277	0.000		
				12870	3	12	0	2326	2341	0.513	99.487	0.128		
				12876	0	19	0	2271	2290	0.830	99.170	0.000		
				12888	1	5	0	2184	2190	0.228	99.772	0.046		
				12951	1	14	0	2000	2015	0.695	99.305	0.050		
				13120	0	10	0	2158	2168	0.461	99.539	0.000		
				13179	1	12	1	2327	2341	0.513	99.487	0.085		
				13197	3	18	0	2376	2397	0.752	99.248	0.125		
				13209	2	11	0	2375	2388	0.461	99.539	0.084		
				13239	1	17	0	2427	2445	0.696	99.304	0.041		
				13287	1	13	0	2560	2574	0.505	99.495	0.039		
				13332	1	15	0	2379	2395	0.627	99.373	0.042		
				13365	1	14	1	2391	2407	0.582	99.418	0.083		
				13405	2	16	0	2648	2666	0.601	99.399	0.075		
				13524	0	12	0	2127	2139	0.561	99.439	0.000		
				13530	0	10	1	2173	2184	0.458	99.542	0.046		
				13551	0	16	0	2176	2192	0.730	99.270	0.000		
				13578	0	11	0	2170	2181	0.504	99.496	0.000		
				13596	3	16	0	2175	2194	0.730	99.270	0.137		
				13609	4	10	0	2147	2161	0.464	99.536	0.185		
				13642	0	8	0	1880	1888	0.424	99.576	0.000		
				13668	1	7	0	1891	1899	0.369	99.631	0.053		
				13702	1	16	1	2002	2020	0.793	99.207	0.099		

				13713	0	11	0	2000	2011	0.547	99.453	0.000		
				13725	2	4	0	1961	1967	0.204	99.796	0.102		
				13758	0	11	1	1703	1715	0.642	99.358	0.058		
				13809	2	8	0	1827	1837	0.436	99.564	0.109		
				13914	2	19	1	2193	2215	0.859	99.141	0.135		
				13939	2	9	1	2152	2164	0.416	99.584	0.139		
				13967	0	12	1	1931	1944	0.618	99.382	0.051		
	protein	ND6	NADH dehydrogenase subunit 6	14159	0	6	0	1093	1099	0.546	99.454	0.000	0.541	0.217
				14225	1	20	0	2129	2150	0.931	99.069	0.047		
				14248	1	12	0	2117	2130	0.564	99.436	0.047		
				14267	0	13	0	1904	1917	0.678	99.322	0.000		
				14383	1	3	0	1549	1553	0.193	99.807	0.064		
				14458	0	11	0	2114	2125	0.518	99.482	0.000		
				14559	4	9	0	2559	2572	0.350	99.650	0.156		
				14568	1	14	0	2550	2565	0.546	99.454	0.039		
	tRNA	TRNE	tRNA-Glu	14680	2	19	0	2487	2508	0.758	99.242	0.080	0.742	0.148
				14684	1	15	0	2580	2596	0.578	99.422	0.039		
				14697	1	19	0	2699	2719	0.699	99.301	0.037		
				14720	1	27	0	2864	2892	0.934	99.066	0.035		
	protein	CYTB	cytochrome b	14759	1	15	1	2504	2521	0.595	99.405	0.079	0.539	0.182
				14803	0	9	0	2156	2165	0.416	99.584	0.000		
				14830	0	12	0	2046	2058	0.583	99.417	0.000		
				14845	0	10	0	2050	2060	0.485	99.515	0.000		
				14860	1	16	0	1914	1931	0.829	99.171	0.052		
				14920	0	10	0	2076	2086	0.479	99.521	0.000		
				14929	0	15	0	2140	2155	0.696	99.304	0.000		
				14944	1	11	0	2219	2231	0.493	99.507	0.045		
				14957	1	12	0	2325	2338	0.513	99.487	0.043		
				14962	1	14	0	2391	2406	0.582	99.418	0.042		
				14984	0	18	0	2478	2496	0.721	99.279	0.000		
				14995	2	15	0	2467	2484	0.604	99.396	0.081		
				15004	2	14	1	2437	2454	0.571	99.429	0.122		
				15040	2	16	0	2404	2422	0.661	99.339	0.083		
				15044	4	21	0	2409	2434	0.864	99.136	0.164		
				15058	1	22	1	2584	2608	0.844	99.156	0.077		
				15090	1	3	0	2713	2717	0.110	99.890	0.037		
				15147	1	9	0	2540	2550	0.353	99.647	0.039		
				15199	2	14	1	2555	2572	0.545	99.455	0.117		
				15275	0	18	0	2639	2657	0.677	99.323	0.000		
				15349	0	15	2	2057	2074	0.724	99.276	0.096		
				15354	0	10	0	2089	2099	0.476	99.524	0.000		
				15391	3	11	0	2012	2026	0.544	99.456	0.148		
				15430	0	12	0	1513	1525	0.787	99.213	0.000		
				15436	1	9	0	1422	1432	0.629	99.371	0.070		
				15499	2	2	10	1393	1407	0.143	99.857	0.853		
				15556	1	9	0	1982	1992	0.452	99.548	0.050		
				15574	0	5	0	2005	2010	0.249	99.751	0.000		
				15590	2	10	0	1934	1946	0.514	99.486	0.103		
				15595	0	9	0	1942	1951	0.461	99.539	0.000		
				15616	0	7	0	1918	1925	0.364	99.636	0.000		
				15698	0	6	0	2344	2350	0.255	99.745	0.000		
				15733	3	12	0	2594	2609	0.460	99.540	0.115		
				15760	1	16	0	2336	2353	0.680	99.320	0.042		
				15811	2	11	1	2166	2180	0.505	99.495	0.138		
	tRNA	TRNT	tRNA-Thr	15926	2	28	0	3317	3347	0.837	99.163	0.060	0.837	
	D-loop			16083	1	13	0	2493	2507	0.519	99.481	0.040	0.547	0.188
				16095	3	9	0	2397	2409	0.374	99.626	0.125		
				16128	1	10	0	2342	2353	0.425	99.575	0.042		
				16328	2	17	0	2259	2278	0.747	99.253	0.088		
				16360	1	12	1	2364	2378	0.505	99.495	0.084		
				16411	1	11	1	2120	2133	0.516	99.484	0.094		
				16427	0	11	0	2132	2143	0.513	99.487	0.000		
				16449	0	10	0	2248	2258	0.443	99.557	0.000		
				16454	0	12	0	2266	2278	0.527	99.473	0.000		
				16495	1	10	0	2467	2478	0.404	99.596	0.040		
				16542	0	3	0	929	932	0.322	99.678	0.000		
				33	1	4	0	974	979	0.409	99.591	0.102		
				61	0	6	0	2017	2023	0.297	99.703	0.000		
				78	1	11	1	2573	2586	0.426	99.574	0.077		
				80	1	13	0	2607	2621	0.496	99.504	0.038		
				91	4	12	2	2793	2811	0.428	99.572	0.213		
				96	0	16	0	2825	2841	0.563	99.437	0.000		
				105	3	16	2	2766	2787	0.575	99.425	0.179		
				120	1	19	0	2700	2720	0.699	99.301	0.037		
				162	2	11	1	2305	2319	0.475	99.525	0.129		
				170	4	19	0	2388	2411	0.789	99.211	0.166		
				186	3	20	0	2564	2587	0.774	99.226	0.116		
				315	0	3	0	1173	1176	0.255	99.745	0.000		
				411	4	13	1	1744	1762	0.740	99.260	0.284		
				498	0	8	0	1292	1300	0.615	99.385	0.000		
				525	0	16	0	1498	1514	1.057	98.943	0.000		
				544	0	14	0	1589	1603	0.873	99.127	0.000		
**Unmethylated lambda DNA**				**Location**	**A call**	**C call**	**G call**	**T call**	**Total**	**% CpG methylated**	**% CpG unmethylated**	**% Seq Error**	**% Methyl Mean**	**% Methyl SD**
				37224	1	37	2	7026	7066	0.524	99.476	0.042	0.554	0.098
				38366	8	41	1	9216	9266	0.443	99.557	0.097		
				28758	4	39	0	7706	7749	0.504	99.496	0.052		
				44220	9	39	1	7856	7905	0.494	99.506	0.127		

				22483	7	55	3	10157	10222	0.539	99.461	0.098		
				43713	7	56	0	8998	9061	0.619	99.381	0.077		
				10226	3	26	1	5666	5696	0.457	99.543	0.070		
				2606	6	47	0	9014	9067	0.519	99.481	0.066		
				2408	8	61	1	11035	11105	0.550	99.450	0.081		
				13204	4	33	2	6549	6588	0.501	99.499	0.091		
				30844	3	48	2	7847	7900	0.608	99.392	0.063		
				16022	0	42	1	6526	6569	0.639	99.361	0.015		
				22221	4	43	1	7590	7638	0.563	99.437	0.065		
				44997	4	46	2	6522	6574	0.700	99.300	0.091		
				9797	3	39	1	7812	7855	0.497	99.503	0.051		
				16674	5	46	2	7127	7180	0.641	99.359	0.097		
				19141	3	49	0	7436	7488	0.655	99.345	0.040		
				46440	6	65	1	9755	9827	0.662	99.338	0.071		
				44129	9	66	0	9882	9957	0.663	99.337	0.090		
				13974	5	27	3	6396	6431	0.420	99.580	0.124		
				19114	6	39	0	7113	7158	0.545	99.455	0.084		
				13337	4	37	0	7004	7045	0.525	99.475	0.057		
				39499	7	40	4	7362	7413	0.540	99.460	0.148		
				36488	2	43	0	8343	8388	0.513	99.487	0.024		
				7492	0	30	1	5636	5667	0.529	99.471	0.018		
				6622	3	50	2	7980	8035	0.623	99.377	0.062		
				17379	2	28	1	6014	6045	0.463	99.537	0.050		
				38216	5	37	4	7653	7699	0.481	99.519	0.117		
				26820	1	35	2	7947	7985	0.438	99.562	0.038		
				32871	5	37	1	6774	6817	0.543	99.457	0.088		
				7139	2	29	0	6349	6380	0.455	99.545	0.031		
				7685	1	41	1	6538	6581	0.623	99.377	0.030		
				35787	5	46	6	8145	8202	0.562	99.438	0.134		
				3780	3	33	1	6657	6694	0.493	99.507	0.060		
				9644	8	40	0	7236	7284	0.550	99.450	0.110		
				39662	4	58	2	8238	8302	0.699	99.301	0.072		
				23630	8	49	0	9223	9280	0.528	99.472	0.086		
				19416	5	53	2	9091	9151	0.580	99.420	0.076		
				27780	5	58	1	8522	8586	0.676	99.324	0.070		
				13197	2	48	1	6587	6638	0.723	99.277	0.045		
				14507	0	21	3	5412	5436	0.387	99.613	0.055		
				44906	3	30	3	7019	7055	0.426	99.574	0.085		
				14465	3	19	1	5037	5060	0.376	99.624	0.079		
				11302	2	34	0	5728	5764	0.590	99.410	0.035		
				43147	10	73	1	12483	12567	0.581	99.419	0.088		
				4010	4	47	1	5448	5500	0.855	99.145	0.091		
				42418	7	74	2	10535	10618	0.698	99.302	0.085		
				3573	5	39	1	7580	7625	0.512	99.488	0.079		
				31890	2	27	10	6710	6749	0.401	99.599	0.178		
				46171	5	60	1	8878	8944	0.671	99.329	0.067		
				1696	7	43	0	8028	8078	0.533	99.467	0.087		
				47046	9	76	4	10721	10810	0.704	99.296	0.120		
				31438	2	35	0	6516	6553	0.534	99.466	0.031		
				46878	6	50	3	9371	9430	0.531	99.469	0.095		
				3505	5	31	1	6219	6256	0.496	99.504	0.096		
				26286	8	51	3	8742	8804	0.580	99.420	0.125		
				36180	11	65	3	11311	11390	0.571	99.429	0.123		
				33760	10	40	1	9449	9500	0.422	99.578	0.116		
				31890	2	27	10	6710	6749	0.401	99.599	0.178		
				26531	7	60	0	8891	8958	0.670	99.330	0.078		
